# Impact of common ALDH2 inactivating mutation and alcohol consumption on Alzheimer’s disease

**DOI:** 10.3389/fnagi.2023.1223977

**Published:** 2023-08-24

**Authors:** Takuya Seike, Che-Hong Chen, Daria Mochly-Rosen

**Affiliations:** Department of Chemical and Systems Biology, Stanford University School of Medicine, Stanford, CA, United States

**Keywords:** Alzheimer’s disease, ALDH2, alcohol, aldehyde, 4-hydroxynonenal, formaldehyde, Alda-1, blood brain barrier

## Abstract

Aldehyde dehydrogenase 2 (ALDH2) is an enzyme found in the mitochondrial matrix that plays a central role in alcohol and aldehyde metabolism. A common ALDH2 polymorphism in East Asians descent (called ALDH2*2 or E504K missense variant, SNP ID: rs671), present in approximately 8% of the world’s population, has been associated with a variety of diseases. Recent meta-analyses support the relationship between this ALDH2 polymorphism and Alzheimer’s disease (AD). And AD-like pathology observed in ALDH2^–/–^ null mice and ALDH2*2 overexpressing transgenic mice indicate that ALDH2 deficiency plays an important role in the pathogenesis of AD. Recently, the worldwide increase in alcohol consumption has drawn attention to the relationship between heavy alcohol consumption and AD. Of potential clinical significance, chronic administration of alcohol in ALDH2*2/*2 knock-in mice exacerbates the pathogenesis of AD-like symptoms. Therefore, ALDH2 polymorphism and alcohol consumption likely play an important role in the onset and progression of AD. Here, we review the data on the relationship between ALDH2 polymorphism, alcohol, and AD, and summarize what is currently known about the role of the common ALDH2 inactivating mutation, ALDH2*2, and alcohol in the onset and progression of AD.

## Introduction

The world population is aging, which is of particular concern because of increase in the number of dementia patients (Khan et al., 2020); one of 5 people over the age of 75 are expected to have Alzheimer’s disease (AD) (Hebert et al., 2013). Patients suffering from dementia, a progressive neurodegenerative disease characterized by cognitive decline, have difficulty living independently and require continuous support from family and healthcare professionals (Lane et al., 2018; Scheltens et al., 2021). As a result, dementia has become a serious problem that not only causes severe suffering to the patients, but also increases the burden of care and medical costs (Wang et al., 2017).

First reported by Alois Alzheimer in 1907 (Alzheimer, 1907), AD is the leading cause of dementia, accounting for 50–75% of all dementia cases (Lane et al., 2018). The neuropathological features of AD include synapse loss, selective neuronal cell death, reduction of certain neurotransmitters, and deposition of abnormal proteins inside and outside neurons (Masters et al., 2015). Various factors have been implicated in the pathogenesis of AD, including abnormal amyloid-β (Aβ) metabolism, tau hyperphosphorylation, oxidative stress, and increases in reactive glia and microglia, the nature of which remains largely unknown (Wang et al., 2017).

Aldehyde dehydrogenase 2 (ALDH2), a member of the ALDH multigene family, is an enzyme found in the mitochondrial matrix that plays a central role in alcohol metabolism (Edenberg and Foroud, 2013). It is involved not only in the metabolism of acetaldehyde generated by alcohol consumption (Klyosov et al., 1996), but also in the metabolism of endogenous and exogenous aldehydes, such as 4-hydroxynonenal (4-HNE), formaldehyde (FA), and malondialdehyde (MDA) (Chen et al., 2019; Rodríguez-Zavala et al., 2019; Dingler et al., 2020; Mu et al., 2021). A well-characterized ALDH2 polymorphism, ALDH2*2, that results in reduced or loss of ALDH2 activity, has attracted attention for its association with various diseases including alcohol flushing (Chen C. H. et al., 2022), malignancy (Zhang and Fu, 2021), cardiovascular disease (Chen et al., 2019), and liver disease (Zhang and Fu, 2021; Seike et al., 2022), and evidence that it is also associated with AD has recently been accumulating.

The effects of alcohol on the human health are clear, as alcohol consumption results in 139 million disability-adjusted life years worldwide (Meza et al., 2022). Despite regional differences, global alcohol consumption is still increasing (Manthey et al., 2019), so is the alarming increase in some alcohol-related diseases and causes of death, such as mental disorders, liver cirrhosis, and cancer (Rumgay et al., 2021). Studies have shown that the ALDH2 polymorphism, ALDH2*2, is protective against alcoholism because it acts in a suppressive manner against excessive alcohol intake due to the discomfort caused by drinking (Edenberg and Foroud, 2013). However, more recent data show that among excessive alcohol consumers, approximately 20% of people carried the ALDH2 deficient polymorphism (Chen et al., 1999; Yokoyama et al., 2002; Brooks et al., 2009). Therefore, the health effect of alcohol consumption in carriers of ALDH2*2 gene cannot be ignored.

The relationship between alcohol consumption and neurodegeneration has been studied for some time (Venkataraman et al., 2017; León et al., 2021; Ramos et al., 2022), and chronic and heavy alcohol consumption may accelerate brain aging and increase the risk of dementia and AD (León et al., 2021; Visontay et al., 2021). What then is the role of alcohol in the pathogenesis of AD under conditions of reduced ALDH2 activity? We have previously shown that daily exposure of ALDH2*2/*2 knock-in mice (E504K missense) to ethanol causes mitochondrial dysfunction, oxidative stress, and increased aldehyde load in the brain (Joshi et al., 2019). In addition, increased AD-related proteins Aβ, phosphorylation of neurofilament tau, and neuroinflammation were also exacerbated in the brains of ethanol-exposed ALDH2*2/*2 knock-in mice compared to wild type (WT) mice (Joshi et al., 2019). If confirmed in humans, these findings strongly suggest that chronic and excessive ethanol consumption, especially among ALDH2*2 carriers, may accelerate the progression and exacerbate the pathogenesis of AD in humans. Recent studies showing that neurofilament light chains, a marker of neuroaxonal injury, are more increased in alcohol dependence patients with the ALDH2 deficient polymorphism provide more support for this hypothesis (Huang et al., 2023).

About 540 million people, of East Asians ancestry, have markedly reduced ALDH2 activity due to a missense mutation in its gene. This indicates that approximately 8% of the world’s population is more vulnerable and susceptible to exposure to toxic acetaldehyde (Brooks et al., 2009). So, will increased global alcohol consumption (Manthey et al., 2019), along with a large ALDH2 polymorphic drinking population, spur an increase in dementia patients? Clarification of this issue could lead to a better understanding of modifiable risk factors for AD. Understand the modifiable risk factors that contribute to the progression of AD pathology together with effective ALDH2 deficiency education campaign may lead to a reduction in the future increase in the number of AD patients. Therefore, it is necessary to piece together fragmentary information, such as the relationship between ALDH2 inactivity and AD and the relationship between alcohol consumption and AD, to understand their influence on the pathogenesis of AD.

## Epidemiological data on ALDH2 polymorphism and Alzheimer’s disease

A cross-sectional community-based study of 690 Koreans aged 65 years and older showed no association between ALDH2*2 and AD (Kim et al., 2004). Another study of 510 Koreans aged 65 years or older observed for 2.4 years also found no significant association between ALDH2*2 and incidence of dementia, AD, or cognitive decline (Shin et al., 2005).

In contrast, genotype frequencies of the ALDH2*2 allele were significantly higher in 447 Japanese AD patients compared with an equal number of sex-, age-, and region-matched non-affected subjects (Ohta et al., 2004). In an analysis of data from 1949 Chinese individuals aged 90 years and older, the ALDH2*2 polymorphism was associated with cognitive dysfunction (Jin et al., 2021). In the Japanese cohort, the frequency of ALDH2*2 was significantly increased in AD subjects compared to the target group, with an odds ratio of 1.41 (Ueno et al., 2022). That study also performed a meta-analysis (1,824 cases, 4,300 controls) including six additional previous case-control studies in Asians (Kamino et al., 2000; Wang et al., 2008; Zhou et al., 2010; Komatsu et al., 2014; Ma and Lu, 2016; Wu Y. Y. et al., 2021) and found that the ALDH2*2 allele was a risk for AD, with an odds ratio of 1.38 (Ueno et al., 2022).

## Natural history of mice with reduced ALDH2 activity

**FIGURE 1:**
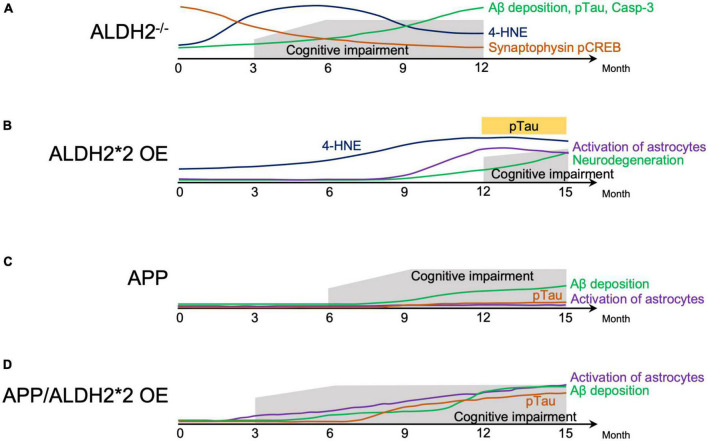
Schematic representation of behavior cellular and molecular changes associated with AD-like symptoms in mice. **(A)** Natural history of factors involved in the pathogenesis of Alzheimer’s disease (AD) in ALDH2^–/–^ mice. **(B)** Natural history of factors involved in the pathogenesis of AD in ALDH2*2 overexpressing (OE) transgenic mice. **(C)** Natural history of factors involved in the pathogenesis of mice expressing AD-associated amyloid precursor protein (APP). **(D)** Natural history of factors involved in the pathogenesis of mice expressing APP, and overexpressing ALDH2*2. 4-HNE, 4-hydroxynon-enal; Aβ, amyloid β; pTau, phosphorylated tau; Casp-3, Caspase 3; pCREB, phosphorylated cyclic-AMP response element binding protein.

Animal models that mimic AD express some of the pathological features of human AD (Akhtar et al., 2022). ALDH2^–/–^ null mice showed progressive cognitive dysfunction from around 3.5–4 months, with AD-like pathological changes, including increased 4-HNE protein adducts in the hippocampus (D’Souza et al., 2015) and cortex (Knopp et al., 2020), Aβ deposition in the brain, tau phosphorylation, increased activated caspase, and defective cAMP-response element binding protein (CREB) signaling (D’Souza et al., 2015; Luo et al., 2016; Knopp et al., 2020). In ALDH2^–/–^ null mice, compared to WT mice, a marked decrease in apical and basal dendritic length, dendritic complexity, and spine density of dorsal hippocampal CA1 pyramidal cells began to be observed around 6 months and was maintained through the age of 12 months. This neuronal degeneration was associated with oxidative stress (Mehder et al., 2020, 2021). The progressive age-related decline in hippocampus-dependent working and spatial memory in ALDH2^–/–^ null mice from around 3.5–4 months (D’Souza et al., 2015) was associated with a decrease in synaptic proteins which were important for learning and memory in the hippocampus (Knopp et al., 2020; Figure 1A).

In transgenic (Tg) mice overexpressing ALDH2*2 (ALDH2*2 OE), aging was accompanied by increased deposition of 4-HNE in the brain, decreased pyramidal cells, increased glial cell activation, and at 12–18 months, tau phosphorylation and learning memory deficits became apparent in the hippocampus (Ohsawa et al., 2008; Figure 1B). In ALDH2*2 OE mice, it took longer for pathological changes to develop compared to ALDH2^–/–^ null mice (D’Souza et al., 2015). Furthermore, in mice where the ALDH2*2 E504K missense mutation was knocked-in (ALDH2*2/*2 KI), increased mitochondrial reactive oxygen species (ROS), Aβ accumulation, and caspase 3 activation at 6 months was observed (These mice are not included in the Figure 1B because of incomplete data) (Joshi et al., 2019). These three mouse models, two mimicking the mutations in human with residual ALDH2 activity of about 2–5%, and the other – with complete absence of ALDH2, show that a reduction in ALDH2 activity alone can mimic AD-like pathology, indicating the importance of reduced ALDH2 activity in AD.

## Acceleration of AD pathology due to decreased ALDH2 activity in mice

Double transgenic (APP/ALDH2*2 OE) mice from a cross between Tg2576 mice expressing a mutant form of human amyloid precursor protein (APP) and DAL mice over expressing the mutant form of ALDH2 (ALDH2*2 OE) showed cognitive dysfunction already at 3 months, accelerated gliosis from 6 months, tau phosphorylation from around 9 months, and Aβ accumulation beginning around 6 months that increased significantly after 12 months (Kanamaru et al., 2015). Pathological changes in APP/ALDH2*2 OE mice were observed earlier and were significantly more pronounced compared to APP mice (Kanamaru et al., 2015), which showed cognitive dysfunction from 6 months (Westerman et al., 2002), Aβ accumulation from 9 to 12 months (Kawarabayashi et al., 2001), no tau phosphorylation (Bilkei-Gorzo, 2014), and no astrocyte activation (Kanamaru et al., 2015; Figures 1C, D). These results indicate an earlier onset of memory impairment and accelerated AD-like pathology in APP/ALDH2*2 OE mice with reduced activity of ALDH2 than in APP mice (Kanamaru et al., 2015). In addition, overexpressing wildtype (active) ALDH2 in APP/PS1 mice (ALDH2 OE/APP/PS1) reduced cognitive dysfunction compared to APP/PS1 mice. This indicates that increased activity of ALDH2 can counteract the pathogenesis of AD (Zhu et al., 2022).

## Relationship between oxidative stress and the pathogenesis of AD in mice

The above animal models suggest that decreased ALDH2 activity increases the pathogenesis of AD and promotes its progression in predisposed individuals (Figure 1) and that increased ALDH2 activity can reduce this pathogenesis. What molecular mechanisms underlie this phenomenon? ALDH2 is an important enzyme responsible for the metabolism of both endogenous and exogenous toxic aldehydes, including 4-HNE, MDA, acetaldehyde and FA (Teng et al., 2001; Chen et al., 2016; Dingler et al., 2020; Li T. et al., 2021; Mu et al., 2021; Kou et al., 2022), and decreased ALDH2 activity is associated with increased vulnerability to oxidative stress (Ohsawa et al., 2003). Indeed, oxidative damage has been reported to precede the appearance of Aβ groups and neurofibrillary tangles in AD patients and different AD animal models (Praticò et al., 2001; Resende et al., 2008), indicating that oxidative stress is an important change that occurs early in AD disease (Keller et al., 2005; Butterfield et al., 2006; Praticò, 2008; Padurariu et al., 2013). In ALDH2^–/–^ null mice (D’Souza et al., 2015; Knopp et al., 2020; Mehder et al., 2021) and ALDH2*2 OE Tg mice (Ohsawa et al., 2008), 4-HNE in the brain is found to be increased early and prior to AD-like symptoms (Figures 1A, B). Therefore, it is necessary to understand the role of toxic aldehydes, such as 4-HNE, acetaldehyde and FA, and decreased ALDH2 activity on AD.

## Effect of 4-HNE on the pathogenesis of AD in humans and mice

4-HNE is elevated in ventricular fluid (Lovell et al., 1997) and brain (Sayre et al., 1997; Markesbery and Lovell, 1998; Williams et al., 2006; Fukuda et al., 2009; Reed et al., 2009) of AD patients. 4-HNE is also increased in the brain tissue of ALDH2^–/–^ null mice (D’Souza et al., 2015; Knopp et al., 2020; Mehder et al., 2021) and ALDH2*2 OE Tg mice (Ohsawa et al., 2008; Figures 1A, B) and in ALDH2*2/*2 KI mice (Joshi et al., 2019). 4-HNE levels in the brain are also increased in senescence accelerated mice P8 (SAMP8), an aging phenotype characterized by memory impairment and behavioral changes (Griñan-Ferré et al., 2016), and in 5XFAD mice, an early-onset transgenic mouse model of AD (Griñán-Ferré et al., 2016; Shin et al., 2020). Interestingly, ALDH2 expression is lower in the hippocampus of SAMP8 at 2 and 9 months relative to age-matched control strain, SAMR1 (Griñan-Ferré et al., 2016), and in the brain of 5XFAD mice at 8 months (Griñán-Ferré et al., 2016) relative to age-matched WT mice. These data indicate that 4-HNE is increased in the context of AD and that one of the reasons for this may be the reduced ALDH2 activity.

The toxicity of 4-HNE is due to changes in cellular function secondary to its ability to readily react with various cellular components with nucleophilic thiol (-SH), amino (-NH2) groups and deoxyguanosine residues, such as deoxyribonucleic acid (DNA) and proteins (Huang et al., 2010; Perluigi et al., 2012; Dalleau et al., 2013). Redox proteomic studies have identified proteins involved in metabolism, cell signaling, pH regulation, neuronal communication, antioxidation and detoxification, neurotransmitter regulation, tau phosphorylation and regulation of APP processing in the context of AD and neurodegeneration (Perluigi et al., 2009; Sultana et al., 2013; Di Domenico et al., 2017), and thus indicating that oxidative modifications by 4-HNE likely play an important role in the pathogenesis of AD.

**FIGURE 2:**
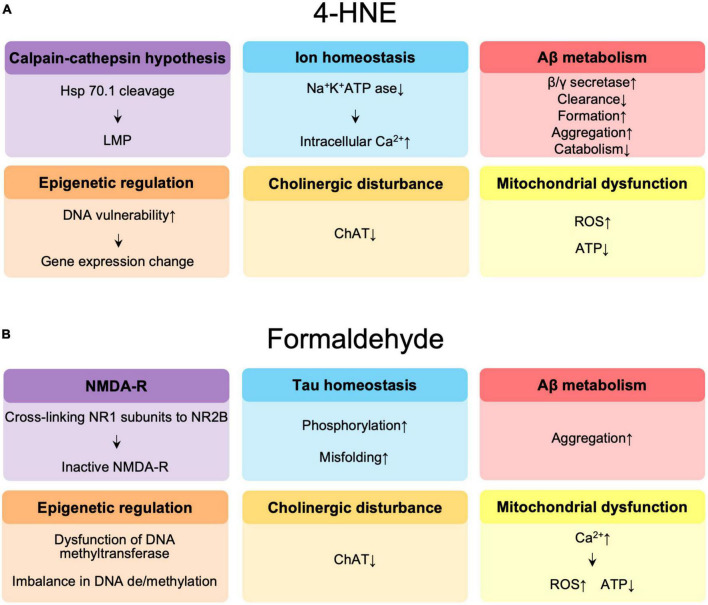
**(A)** Summary of the effects of 4-hydroxynonenal (4-HNE) in the pathogenesis of Alzheimer’s disease (AD). **(B)** Summary of the effects of formaldehyde in the pathogenesis of AD. Hsp 70.1, heat shock protein 70.1; LMP, lysosomal membrane permeabilization; Aβ, amyloid β; ChAT, choline acetyltransferase; ROS, reactive oxygen species; ATP, adenosine triphosphate; NMDAR, N-methyl-D-aspartate receptor.

The molecular mechanisms by which 4-HNE is involved in the pathogenesis of AD are summarized in Figure 2A. The reduced DNA binding ability of histones and altered chromatin structure due to oxidative modification of 4-HNE increases the vulnerability of DNA to oxidation in the brains of AD patients (Drake et al., 2004) and may affect transcription, thus leading to accelerated aging and neurodegeneration (Bohr, 2002). 4-HNE leads to dysfunction of glucose and glutamate transporters, mitochondrial dysfunction, and reduced adenosine triphosphate (ATP) levels, and is involved in synaptic degeneration (Keller et al., 1997). Decrease in neuronal Na^+^/K^+^ATPase activity and increase in intracellular free calcium concentration by 4-HNE results in increased neuronal vulnerability (Mark et al., 1997). In addition, 4-HNE-induced lysosomal membrane disruption by cleavage of heat shock protein 70.1 is involved in neuronal degeneration (Yamashima et al., 2020, 2022, 2023). Decreased activity of choline acetyltransferase by 4-HNE has been implicated in memory impairment (Bruce-Keller et al., 1998). 4-HNE enhances the production of Aβ through increased activity of γ-secretase (Gwon et al., 2012) and β-secretase (Tamagno et al., 2002, 2005), and also forms an adduct with neprilysin, an amyloid-degrading enzyme, reducing enzyme activity and Aβ turnover (Wang et al., 2003). Finally, changes in Aβ disposition through increased Aβ formation (Liu et al., 2008), aggregation (Chen et al., 2006; Siegel et al., 2007), decreased catabolism (Wang et al., 2009), and clearance (Shringarpure et al., 2000; Owen et al., 2010) are mediated by 4-HNE.

## Effect of formaldehyde on the pathogenesis of AD

FA is elevated in the brains (He et al., 2010; Tong et al., 2011) and urine (Chen F. et al., 2022) of AD patients, in the brains of aged rats (Tong et al., 2013b), APP/PS1 mice (Yue et al., 2019) that expresses APP and a mutant human presenilin 1 (Lok et al., 2013), and in ALDH2^–/–^ null mice (Tan et al., 2018; Ai et al., 2019). Administration of FA to adult rats mimicked age-related memory decline in aging rats (Tong et al., 2013a; Tan et al., 2018). Administration of an inhibitor (Daidzin) of ALDH2 to rats increased hippocampal FA levels (Tong et al., 2013b). The accumulation of FA in the brain of ALDH2^–/–^ null mice was associated with hyperglycemia and cognitive impairment (Tan et al., 2018). Together, these results suggest that the accumulation of FA in the brain leads to cognitive dysfunction, and that reduced ALDH2 activity is involved in the accumulation of FA in the brain.

The possible molecular mechanisms by which FA is involved in the pathogenesis of AD are summarized in Figure 2B. FA may contribute to age-related cognitive decline by impairing DNA methyltransferase function (Tong et al., 2015), causing an imbalance (Miller et al., 2010) between DNA methylation and demethylation, a critical step in memory formation (Tong et al., 2013a; Li T. et al., 2021). FA acts synergistically with Aβ to promote ROS generation by enhancing calcium influx, it inhibits cyclooxygenase activity, and decreases ATP production, the amount of coenzyme Q10 in mitochondria, resulting in neuronal cell death (Kou et al., 2022). FA also reduces acetylcholine levels by inhibiting choline acetyltransferase (Zhang et al., 2019). N-methyl-D-aspartate receptors (NMDAR), which are composed of NR1 and NR2 or NR3 subunits, play an important role in learning and memory as well as excitatory neurotransmission and synaptic plasticity (Shimizu et al., 2000). Significant reductions in NR1 and NR2 expression have been observed in the hippocampus of aging rats (Clayton and Browning, 2001; Clayton et al., 2002; Mesches et al., 2004) and in AD patients (Hynd et al., 2004; Amada et al., 2005). FA has been reported to be involved in learning and memory control by inactivating NMDAR via NR1/NR2B, thereby suppressing hippocampal long-term potentiation (Tong et al., 2013b; Ai et al., 2019). Excess FA induces hyperphosphorylation of tau (Lu et al., 2013) and misfolding of tau protein via glycogen synthase kinase-3β (GSK-3β) (Elyaman et al., 2002), an important tau kinase, to form spherical amyloid-like aggregates with high cytotoxicity (Nie et al., 2007a,b). FA is a very reactive cross-linking agent in Aβ aggregation that promotes the formation of Aβ dimers, oligomers, and fibrils by cross-linking K28 (lysine, K) residues in the β-turn of the Aβ monomer (Chen et al., 2006; Kou et al., 2022). The accumulation of Aβ in APP/PS1 mice is accompanied by a progressive increase in cortical FA levels, suggesting that FA may promote Aβ accumulation (Yue et al., 2019).

## Possible improvement of cognitive dysfunction through activation of ALDH2

As discussed above, decreased ALDH2 activity is involved in the pathogenesis of AD via various pathways associated with aldehyde accumulation and regardless of the cause of pathogenesis, increased activity of ALDH2 appears to correlate with amelioration of the pathogenesis. For example, elevated level of homocysteine is an independent risk factor for AD (Seshadri et al., 2002; Van Dam and Van Gool, 2009; Hu et al., 2016) and exposure of homocysteine to hippocampus of rats, induces learning and memory impairment (Zhang et al., 2009; Li et al., 2014). However, hydrogen sulfide, which induced an increase in ALDH2 expression, reduced the accumulation of reactive aldehydes in the brain and improved cognitive impairment in these rats (Li et al., 2017). Another example is the correlation between elevated FA and AD pathology in 3XFAD mice as compared to control mice with aging. This elevation in FA levels was associated with reduced ALDH2 activity in the brain, and activation of ALDH2 with Alda-1, a small molecule enzyme activator (Chen et al., 2008), significantly reduced brain FA levels and improved neurological dysfunction (Tao et al., 2022). Similarly, overexpression of the ALDH2 gene significantly improved cognitive function in APP/PS1 AD mice (Yang et al., 2021) and reduced hyperglycemia and improved cognitive function by reducing FA in a mouse model of diabetes (Tan et al., 2018). Rat hippocampal neurons overexpressing ALDH2 by gene transfer showed resistance to 4-HNE-induced neurite damage, decreased caspase-3 protein expression, decreased ROS levels, and decreased disruption of mitochondrial transmembrane potentials (Bai and Mei, 2011).

Further studies showed that treatment with Alda-1, which activates ALDH2 (Chen et al., 2008), reduced the Aβ-induced increase in 4-HNE, mitochondrial dysfunction, and decreased ATP in HT22 cells (Yang et al., 2021). Treatment of mouse microglial BV2 cells or mouse neuronal cells (Neuro-2a) with hydrogen peroxide to increase oxidative stress, resulted in increased FA, decreased ALDH2 activity, and increased pro-apoptotic protein, B-cell lymphoma-2-associated X (BAX), and all of these were ameliorated by treatment with Alda-1 (Tao et al., 2022). Furthermore, increased aldehydic load, oxidative stress, reduced ATP levels and increased mitochondrial dysfunction seen in fibroblasts of an AD patient that also has an ALDH2*2 mutation or overexpression of ALDH2*2 in fibroblasts derived from AD patients with ApoE ε4 allele relative to healthy subjects, were all reduced following treatment with Alda-1 (Joshi et al., 2019). Taken together, these data suggest that activation of ALDH2 is neuroprotective and may prevent cognitive dysfunction and that having reduced ALDH2 activity may render the brain more vulnerable to cognitive function decline.

## Epidemiological data of Alzheimer’s disease and alcohol exposure

A meta-analysis of 14,646 AD patients (Anstey et al., 2009) and a systematic review of 45 studies on the epidemiology of alcohol consumption and risk of dementia or cognitive decline (Ilomaki et al., 2015) indicated that the risk of AD was reduced in light to moderate drinkers compared to non-drinkers. However, a number of studies, as reviewed below, have shown that alcohol consumption, especially heavy amounts, is a risk for dementia. An observational study conducted in the United Kingdom (UK) over a 30-year period revealed that moderate or higher alcohol consumption increased the risk of developing hippocampal atrophy (Topiwala et al., 2017). An analysis of 13,342 individuals aged 40 to 73 years using UK Biobank data showed that alcohol consumption exceeding 1 drink per day was associated with a significant cognitive decline (Piumatti et al., 2018). Another analysis of 397 dementia cases from a UK study involving 9,087 participants aged 35–55 years with an average of 23-year follow ups showed that alcohol consumption of 14 drinks or more per week was a risk for dementia (Sabia et al., 2018). In 2019, an observational study of 3,021 older United States adults with a median age of 78 years and a median follow-up of 6 years showed that drinking more than 14 drinks per week was associated with lower cognitive scores (Koch et al., 2019). A cohort study of 19,887 middle-aged and older U.S. adults with a mean age of 61.8 years in 2020 reported that heavy drinking was associated with risk of dementia. However, drinking 10–14 drinks per week resulted in better cognitive function (Zhang et al., 2020). As for wine, it was reported that mild to moderate consumption of wine reduced the risk of cognitive decline, but excessive consumption increased the risk of dementia due to direct neurotoxic effects (Reale et al., 2020). Based on these results, excessive alcohol consumption (>21 drinks/week) was recognized as a new risk factor for dementia (Livingston et al., 2020).

In addition, several recent dose-response meta-analyses have shown that alcohol intake of 27.5 g/day (about two alcoholic beverages) or more is associated with an increased risk of progression to dementia in people with mild cognitive impairment (Lao et al., 2021), moderate drinking of less than 11 g/day (Ran et al., 2021) or 12.5 g/day (Xu et al., 2017) was associated with a reduced risk of dementia, and excessive drinking of 38 g/day or more was associated with an increased risk (Xu et al., 2017). In a 2023 report from South Korea analyzing 100,292 people with a diagnosis of dementia and 6.3 years of follow-up among 3,933,382 people with a mean age of 55 years, alcohol consumption of 30 g/day or more was also associated with an increased risk of dementia (Jeon et al., 2023; Table 1). Unfortunately, the status of ALDH2 genotyping of these studies is unknown.

**TABLE 1:** Relationship between alcohol consumption and risk of dementia.

References	Number of subjects	Pure alcohol equivalent (gram/day)	Standard drink (1 drink = 14 gram pure alcohol/day)	Risk of dementia
Ran et al., 2021	131777 (meta-analysis of 16 studies)	11>	0.8 drink>	↓
Xu et al., 2017	70150 (meta-analysis of 10 studies)	12.5>	0.9 drink>	↓
Lao et al., 2021	4244 (meta-analysis of 6 studies)	27.5<	2.0 drink<	↑*
Jeon et al., 2023	3933382 (retrospective cohort study)	30<	2.1 drink<	↑
Xu et al., 2017	70150 (meta-analysis of 10 studies)	38<	2.7 drink<	↑

>means “less than.” <means “more than.” Drink equivalents are rounded to the nearest 0.1.

*Increased risk of progression of dementia in patients with mild cognitive impairment.

In relation to alcohol use disorder (AUD), an analysis of 57,353 early-onset dementia patients in a French population showed that 56.6% were associated with alcohol-related brain damage or AUD (Schwarzinger et al., 2018). Analyses of 129,182 patients with AUD in the United States (Zhang et al., 2022) and 13,568 patients with dementia in Western Austria (Zilkens et al., 2014) similarly showed that AUD is a risk for AD (Zhang et al., 2022).

Alcohol consumption is generally considered a modifiable risk factor for dementia, but results in the literature are not entirely consistent (Wiegmann et al., 2020). The dependence of drinking behavior on sociocultural context and the involvement of health-related factors complicates the analysis for drinking (Livingston et al., 2020). However, the effects of chronic heavy drinking on brain are clear (Visontay et al., 2021), demonstrating that excessive drinking causes alcohol-related brain damage.

## Effect of alcohol on Alzheimer’s disease and neurodegeneration

**FIGURE 3:**
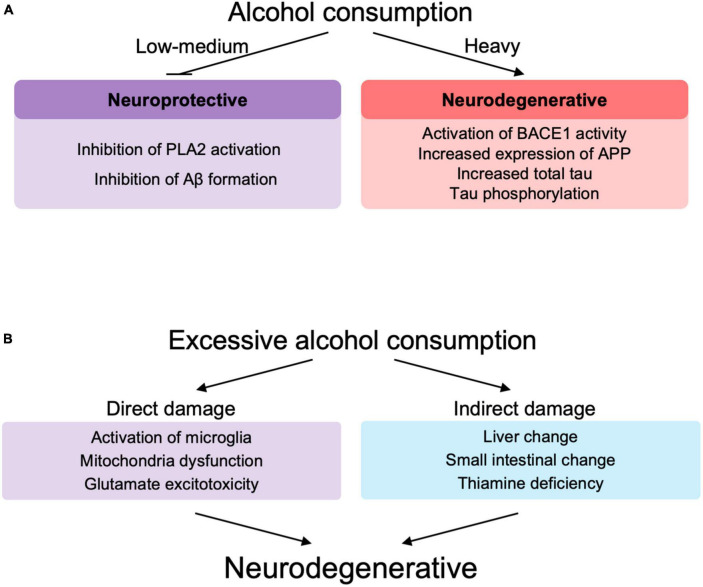
**(A)** The role of alcohol at different levels of drinking in neurodegeneration. **(B)** Direct and indirect role of excessive alcohol consumption in neurodegeneration. PLA2, phospholipase A2; Aβ, amyloid β; BACE-1, beta-site amyloid precursor protein cleaving enzyme-1; APP, amyloid precursor protein.

In support of these epidemiological data, *in vitro* and animal studies also showed alcohol concentration-dependent toxic effects of Aβ (Figure 3A). Low concentrations of alcohol (0.02–0.08%) inhibited calcium-sensitive activation of cytosolic phospholipase A2 induced by Aβ and α-synuclein (related to synaptic damage) (Bate and Williams, 2011). Low concentrations of alcohol (10 mM), equivalent to low to moderate alcohol drinking disrupted the salt bridge between Asp23 and Lys28, which is required for amyloid dimerization, and inhibited Aβ formation (Ormeño et al., 2013). The amyloid pore/channel hypothesis suggests that low concentration of alcohol could inhibit amyloid aggregation, thereby prevented increased calcium concentrations in neurons, and neuronal degeneration, and may explain some of the protective effects from consumption of low amounts of alcoholic beverages (Parodi et al., 2015). Thus, alcohol consumption at low or moderate concentrations may have a protective effect on Aβ toxicity to neurons (Bate and Williams, 2011; Ormeño et al., 2013; Peng et al., 2020) (These observations, however, were not made in the context of ALDH2 deficiency.) On the other hand, excessive amounts of alcohol lead to increased BACE1 activity with increased expression of presenilin and nicastrin, which are involved in the production of Aβ, and APP (Kim et al., 2011). In addition, excessive amounts of alcohol are also responsible for an increase in total tau due to decreased phosphorylation of proteins associated with the mTOR/AKT pathway (Hoffman et al., 2019; Kamal et al., 2020), increased expression of cyclin-dependent kinase 5 (Rajgopal and Vemuri, 2001) and GSK3β (Hoffman et al., 2019) that are involved in tau hyperphosphorylation.

Excessive alcohol consumption may be involved in the decline of brain functions and accelerated neurodegeneration (Venkataraman et al., 2017; León et al., 2021; Ramos et al., 2022; Figure 3B). Excessive chronic alcohol consumption leads to neuroinflammation and degeneration via impaired mitochondrial energy production/dynamics (León et al., 2021) and glutamatergic excitotoxicity (Kamal et al., 2020) as well as microglial activation (Crews et al., 2017; Venkataraman et al., 2017; Asatryan et al., 2018; Ramos et al., 2022) through Toll-like receptors (TLRs), nod-like receptor family pyrin domain containing 3 inflammasomes, cytokines, and purinergic P2X receptors. Microglial activation leads to elevated peripheral macrophage infiltration, elevated immune mediators, elevated cytokine chemokine release and extracellular vesicles, which are associated with neurodegeneration and synapse loss (Ramos et al., 2022). TRL4 signaling by alcohol and Aβ fibril may also play an important role in microglial activation (Venkataraman et al., 2017). Similar to the involvement of immune cells in lifestyle diseases such as non-alcoholic fatty liver disease (Seike et al., 2020, 2021), immune abnormalities also play an important role in AD, since alcohol appears to modify the pathogenesis of AD via immune dysfunction (Wu K. M. et al., 2021). Chronic alcohol consumption induces glutamatergic excitotoxicity by producing elevated glucocorticoid and glutamate concentrations. This glucocorticoid-induced increase in NMDAR and its subunits, NR2A and NR2B, leads to calcium influx into neurons, forming BAX pores on mitochondria, releasing cytochrome c and inducing neuronal cell apoptotic death (Kamal et al., 2020).

Alcohol is also involved in indirect neuroinflammation by increasing the expression of inflammatory cytokines, endothelial prostaglandins and inducible nitric oxide synthase, in part through thiamine deficiency, small intestine and liver changes, withdrawal symptoms and traumatic brain injury (Venkataraman et al., 2017; Figure 3B). Based on these studies, alcohol-induced neuroinflammation may play an important role in the pathogenesis of AD, since alcohol can cause neuroinflammation in AD (Venkataraman et al., 2017; León et al., 2021; Díaz et al., 2022; Ramos et al., 2022).

## Effect of alcohol on blood brain barrier

The blood brain barrier (BBB) is formed by endothelial cells and the pericytes and astrocytes that surround them between the blood and the central nervous system and plays an important role in maintaining homeostasis of brain structure and function (Obermeier et al., 2013). Various dysfunctions of the BBB in AD are thought to be involved (Sweeney et al., 2018; Andjelkovic et al., 2023). The intricate structure of BBB makes it susceptible to dysfunction (Banks, 2016), and excess alcohol exposure is one of the factors that reduces BBB stability. The presence or absence of BBB dysfunction in the pathogenesis of alcohol and AD is important in considering the effects of peripheral Aβ on the brain, immune cell infiltration, and involvement of fluid factors in the brain.

Intraperitoneal (Eisele et al., 2010) and intravenous (Morales et al., 2020) administration of Aβ-containing brain extracts from aged APP Tg mice to young allogeneic mice resulted in deposition of Aβ in the meninges and brain. And C57BL/6J mice genetically modified to synthesize human Aβ only in the liver have been reported to have increased plasma and brain Aβ (Lam et al., 2021). On the other hand, intraperitoneal administration of ^13^C-isotope-labeled brain extracts from mice expressing human Aβ to APP Tg mice resulted in long-term detection of injected Aβ in liver and lymphoid tissue but not in brain (Brackhan et al., 2022). It remains unclear what effect peripheral Aβ has on Aβ deposition in the brain. However, it is common to observe in patients with dementia a mixed pathology with multiple brain lesions (Schneider et al., 2007), and AD patients often experience multiple “hits” which are thought to contribute to a more rapid cognitive decline toward dementia (Zhu et al., 2004; Chakrabarti et al., 2015). It appears likely that alcohol-induced BBB impairment could be one of these hits, and indeed, chronic alcohol consumption has been reported to directly decrease BBB stability and increase the influx of inflammatory mediators into the brain parenchyma (Singh et al., 2007; Pimentel et al., 2020; Ramos et al., 2022).

**FIGURE 4:**
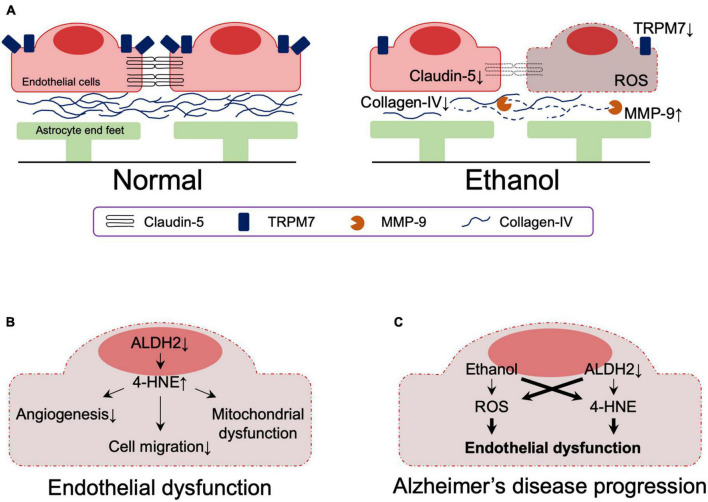
**(A)** Effects of ethanol on the blood-brain barrier. **(B)** The effect of reduced ALDH2 activity on the blood-brain barrier. **(C)** Effects of ethanol and reduced ALDH2 activity on the integrity of the blood brain barrier. Thick lines indicate pathways of exacerbation. MMP-9, matrix metallopeptidase 9; TRPM7, transient receptor potential melastatin-subfamily member 7; ROS, reactive oxygen species; 4-HNE, 4-hydroxynonenal.

Analysis of postmortem samples from alcoholics without liver cirrhosis or nutritional deficiencies showed that the expressions of the tight junction (TJ) protein, claudin-5, and the basement membrane protein, collagen-IV, were significantly reduced in the dorsolateral prefrontal cortex, resulting in reduced BBB integrity (Rubio-Araiz et al., 2017). This observation may be related to increased activity of matrix metallopeptidase 9, which is capable of degrading extracellular matrix components and is associated with impaired basement membrane, and impaired angiogenesis (Abdul Muneer et al., 2012; Rubio-Araiz et al., 2017). It has also been reported that alcohol-induced downregulation of transient receptor potential melastatin-subfamily member 7 expression caused BBB dysfunction as a result of loss of endothelial cell integrity (Chang et al., 2018; Figure 4A). *In vitro* studies using primary human brain microvascular endothelial cells also showed that alcohol-induced oxidative stress led to activation of myosin light chain (MLC) kinase, phosphorylation of MLC and TJ proteins, decreased BBB integrity, and accelerated monocyte migration through the BBB (Haorah et al., 2005).

## Effect of ALDH2 inactivity and alcohol on blood brain barrier

A role of ALDH2 in BBB integrity has also been suggested. ALDH2 is involved in oxidative and reductive reactions in vascular endothelial cells (Nannelli et al., 2020) and reduced ALDH2 activity is associated with the acquisition of an early aging phenotype of endothelial cells (Nannelli et al., 2018), atherosclerosis (Cai et al., 2023), and coronary artery disease (Xu et al., 2011; Yasue et al., 2019). Reduced ALDH2 activity increases 4-HNE-medicated reduction of anti-angiogenesis (Roy and Palaniyandi, 2020) and migration (Roy et al., 2020) in mouse coronary endothelial cells and is involved in 4-HNE-induced endothelial dysfunction and mitochondrial dysfunction in human umbilical vein endothelial cells (Nannelli et al., 2018; Figure 4B).

Although ethanol has a dose-dependent protective effect against endothelial cell senescence by activating ALDH2, decreased ALDH2 activity in endothelial cells impairs this protection by ethanol (Xue et al., 2018, 2019). Furthermore, studies using induced pluripotent stem cell-derived endothelial cells demonstrated that even minimal amounts of alcohol impair vascular endothelial function in the presence of ALDH2*2 (Guo et al., 2023).

Together, these results indicate that reduced ALDH2 activity leads to endothelial dysfunction, which is exacerbated by ethanol exposure. Although their involvement in the context of AD has not yet been directly determined, endothelial dysfunction due to reduced ALDH2 activity and its exacerbation by ethanol likely contribute to the pathogenesis of AD (Figure 4C).

## Role of ALDH2*2 and alcohol in the pathogenesis of AD

**FIGURE 5:**
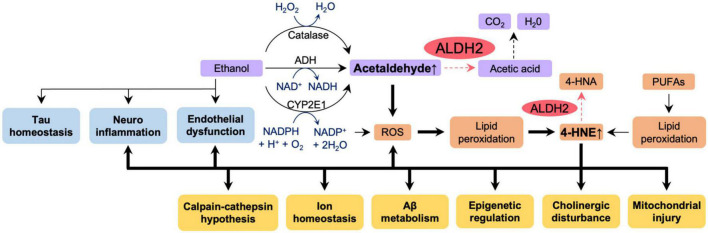
The effect of reduced ALDH2 activity and ethanol on AD pathology. Ethanol appears to affect directly tau homeostasis, increase neuroinflammation and endothelial dysfunction – all pathologies associated with AD (blue boxes). Ethanol metabolism (purple boxes) results in accumulation of acetaldehyde. Accumulation of acetaldehyde decreases activity of ALDH2 and exacerbates ROS production, lipid peroxidation and accumulation of other toxic aldehydes, such as 4HNE (orange boxes). All these further increase AD-associated pathologies (yellow boxes). Thick lines indicate pathways of exacerbation and dotted lines indicate pathways of attenuation. In humans and in knock-in mice, ALDH2 activity in wild type, ALDH2*1/*1, is 100%; ALDH2 activity in ALDH2*2/*1 (heterozygotes) is 10–45%; ALDH2 activity in ALDH2*2/*2 (homozygotes) is 2–5%. Carriers of ALDH2*2/*2 accumulate more aldehydes as indicated by the dotted pathway is the reduced ALDH2 activity. 4-HNE, 4-hydroxynonenal; 4-HNA, 4-hydroxynonenic acid; ROS, reactive oxygen species; PUFAs, polyunsaturated fatty acids; Aβ, amyloid β.

Our laboratory has previously shown that fibroblasts from AD patients with ALDH2*2 mutation or with ApoE ε4 allele overexpressing ALDH2*2 exhibit increased aldehydic load, oxidative stress, and mitochondrial dysfunction when compared to fibroblasts from healthy individuals, and that ethanol exposure further aggravated these dysfunctions (Joshi et al., 2019). Mitochondrial dysfunction, oxidative stress, and elevated brain aldehyde levels were also observed in ethanol-exposed WT mice, but these changes were more pronounced in ALDH2*2/*2 KI mice. The increased aldehydic load in WT mice treated with 1g/kg/day ethanol for 11 weeks and the subsequent aldehydic adducts on mitochondrial proteins results in mitochondrial dysfunction, and the reduced ALDH2 activity in ALDH2*2/*2 KI mice further exacerbates this mechanism by reducing the clearance of toxic aldehydes. We found that chronic ethanol exposure resulted in increased the levels of the AD-related protein Aβ and neuroinflammation in the brains of ALDH2*2/*2 KI mice compared to WT mice (Joshi et al., 2019). Importantly, all these pathological processes are reversed by Alda-1 treatment suggested that reduced activity of ALDH2 has a major role in mitochondrial dysfunction and in AD pathology in AD patient-derived cells and in mouse models (Joshi et al., 2019). These data indicate increased vulnerability of neurons to oxidative stress by alcohol in the presence of inactive, ALDH2*2, enzyme relative to functional ALDH2 and thus suggest that alcohol consumption may exacerbate the pathogenesis of AD patients in carriers of ALDH2*2. Thus, as depicted in Figure 5, we can now link data on the relationship between ALDH2 polymorphisms and AD risk, oxidative stress and AD, aldehydes and AD, alcohol and BBB integrity, and ALDH2 and BBB integrity, as shown in Figures 2A, 3, 4A, B. Importantly, a more detailed mechanistic hypothesis of the contribution of alcohol consumption to the pathological progression of AD in the reduction of ALDH2 activity emerges (Figure 5). We highlighted the role of 4-HNE accumulation in this mechanism, but recognize that other pathways, yet to be identified, may also contribute to the negative impact of alcohol consumption in patients at risk for AD.

Clearly, the effects of alcohol on AD are related to the amount, pattern, frequency and the type of the alcohol consumed, the nutritional status of the individual, and the genotype of the individual (Peng et al., 2020). A study of AD patients from Taiwan found no relationship between ALDH2 polymorphisms, alcohol consumption, and AD (Wu Y. Y. et al., 2021). But caution should be exercised in interpreting the results, as the study relied only self-reported alcohol use and made no mention of the amount of alcohol consumed. Since the ALDH2*2 mutation causes a 5-fold increase in acetaldehyde after moderate alcohol drinking compared to wild-type ALDH2 (Yokoyama et al., 2008), and increased acetaldehyde levels last much longer (Chen et al., 2009), the effects of ethanol consumption on the human body cannot be ignored, especially in ALDH2*2 individuals. It is likely that this elevated aldehyde toxicity may be a contributor to the pathogenesis of AD. It is therefore important that future large-scale epidemiological studies that include alcohol consumption data should be conducted to clarify the roles of ALDH2 polymorphism and whether a smaller amount of alcohol consumption can contribute to AD in individuals carrying the ALDH2*2 variant.

Also of interest may be the role of ALDH1A1 in AD, an enzyme involved in alcohol-induced facial flushing, alcohol sensitivity and dependence in Caucasians (Yoshida et al., 1989; Spence et al., 2003), and in acetaldehyde detoxification (Marchitti et al., 2008). Recently, the relationship between ALDH1A1 and AD has been reported (Nikhil et al., 2019; Li X. et al., 2021; Tandon et al., 2023). Both epidemiological studies in AD patients, determining ALDH1A1 genotype and alcohol consumption, and mechanistic studies in mice with such mutations will allow a better assessment of the potential risk of alcohol consumptions for AD development in subjects with ALDH1A1 insufficiency.

## Conclusion

Epidemiological studies of AD patients and analyses of ALDH^–/–^ null mice, ALDH2*2 OE Tg mice (Figure 1) and ALDH2*2/*2 KI mice, all suggest that ALDH2 dysfunction may be a risk factor for AD, and that ethanol-induced aldehyde accumulation and its toxicity due to decreased ALDH2 activity may be a factor in AD pathogenesis. Ethanol exposure, which increases mitochondrial dysfunction, neuroinflammation and neurodegeneration and causes BBB disruption, exacerbates AD-like pathology in ALDH2*2/*2 KI mice. Therefore, a detailed large-scale epidemiological evaluation of how alcohol consumption and ALDH2 genotype affect AD and AD progression is needed. Since alcohol consumption is a modifiable lifestyle choice, clear understanding of ALDH2, alcohol consumption and AD onset and progression may help to reduce the number of AD patients, especially in the approximately 8% of the world population with ALDH2*2 polymorphism.

## Author contributions

TS: conceptualization, data curation, writing–original draft preparation, and visualization. TS and C-HC: investigation. C-HC and DM-R: writing–review and editing. DM-R: project administration. All authors contributed to the article and approved the submitted version.

## References

[B1] Abdul Muneer P. M. Alikunju S. Szlachetka A. M. Haorah J. (2012). The mechanisms of cerebral vascular dysfunction and neuroinflammation by MMP-mediated degradation of VEGFR-2 in alcohol ingestion. *Arteriosc. Thromb. Vasc. Biol*. 32 1167–1177. 10.1161/atvbaha.112.247668 22402362 PMC3501346

[B2] Ai L. Tan T. Tang Y. Yang J. Cui D. Wang R. (2019). Endogenous formaldehyde is a memory-related molecule in mice and humans. *Commun. Biol*. 2:446. 10.1038/s42003-019-0694-x 31815201 PMC6884489

[B3] Akhtar A. Gupta S. M. Dwivedi S. Kumar D. Shaikh M. F. Negi A. (2022). Preclinical models for Alzheimer’s disease: Past, present, and future approaches. *ACS Omega* 7 47504–47517. 10.1021/acsomega.2c05609 36591205 PMC9798399

[B4] Alzheimer A. (1907). Uber eine eigenartige Erkrankung der Hirnrinde. *Zentralbl. Nervenh. Psych.* 18 177–179.

[B5] Amada N. Aihara K. Ravid R. Horie M. (2005). Reduction of NR1 and phosphorylated Ca2+/calmodulin-dependent protein kinase II levels in Alzheimer’s disease. *Neuroreport* 16 1809–1813. 10.1097/01.wnr.0000185015.44563.5d 16237332

[B6] Andjelkovic A. V. Situ M. Citalan-Madrid A. F. Stamatovic S. M. Xiang J. Keep R. F. (2023). Blood-brain barrier dysfunction in normal aging and neurodegeneration: Mechanisms, impact, and treatments. *Stroke* 54 661–672. 10.1161/strokeaha.122.040578 36848419 PMC9993074

[B7] Anstey K. J. Mack H. A. Cherbuin N. (2009). Alcohol consumption as a risk factor for dementia and cognitive decline: Meta-analysis of prospective studies. *Am. J. Geriatr. Psychiatry* 17 542–555. 10.1097/JGP.0b013e3181a2fd07 19546653

[B8] Asatryan L. Ostrovskaya O. Lieu D. Davies D. L. (2018). Ethanol differentially modulates P2X4 and P2X7 receptor activity and function in BV2 microglial cells. *Neuropharmacology* 128 11–21. 10.1016/j.neuropharm.2017.09.030 28943285 PMC6492934

[B9] Bai J. Mei Y. (2011). Overexpression of aldehyde dehydrogenase-2 attenuates neurotoxicity induced by 4-hydroxynonenal in cultured primary hippocampal neurons. *Neurotox Res*. 19 412–422. 10.1007/s12640-010-9183-1 20361289

[B10] Banks W. A. (2016). From blood-brain barrier to blood-brain interface: New opportunities for CNS drug delivery. *Nat. Rev. Drug Discov*. 15 275–292. 10.1038/nrd.2015.21 26794270

[B11] Bate C. Williams A. (2011). Ethanol protects cultured neurons against amyloid-β and α-synuclein-induced synapse damage. *Neuropharmacology* 61 1406–1412. 10.1016/j.neuropharm.2011.08.030 21903110

[B12] Bilkei-Gorzo A. (2014). Genetic mouse models of brain ageing and Alzheimer’s disease. *Pharmacol. Ther*. 142 244–257. 10.1016/j.pharmthera.2013.12.009 24362083

[B13] Bohr V. A. (2002). Repair of oxidative DNA damage in nuclear and mitochondrial DNA, and some changes with aging in mammalian cells. *Free Radic. Biol. Med*. 32 804–812. 10.1016/s0891-5849(02)00787-6 11978482

[B14] Brackhan M. Calza G. Lundgren K. Bascuñana P. Brüning T. Soliymani R. (2022). Isotope-labeled amyloid-β does not transmit to the brain in a prion-like manner after peripheral administration. *EMBO Rep*. 23:e54405. 10.15252/embr.202154405 35620875 PMC9253763

[B15] Brooks P. J. Enoch M. A. Goldman D. Li T. K. Yokoyama A. (2009). The alcohol flushing response: An unrecognized risk factor for esophageal cancer from alcohol consumption. *PLoS Med*. 6:e50. 10.1371/journal.pmed.1000050 19320537 PMC2659709

[B16] Bruce-Keller A. J. Li Y. J. Lovell M. A. Kraemer P. J. Gary D. S. Brown R. R. (1998). 4-Hydroxynonenal, a product of lipid peroxidation, damages cholinergic neurons and impairs visuospatial memory in rats. *J. Neuropathol. Exp. Neurol*. 57 257–267. 10.1097/00005072-199803000-00007 9600218

[B17] Butterfield D. A. Reed T. Perluigi M. De Marco C. Coccia R. Cini C. (2006). Elevated protein-bound levels of the lipid peroxidation product, 4-hydroxy-2-nonenal, in brain from persons with mild cognitive impairment. *Neurosci. Lett*. 397 170–173. 10.1016/j.neulet.2005.12.017 16413966

[B18] Cai N. Li C. Gu X. Zeng W. Liu J. Zeng G. (2023). ALDH2 rs671 and MTHFR rs1801133 polymorphisms are risk factors for arteriosclerosis in multiple arteries. *BMC Cardiovasc. Disord*. 23:319. 10.1186/s12872-023-03354-0 37355582 PMC10290786

[B19] Chakrabarti S. Khemka V. K. Banerjee A. Chatterjee G. Ganguly A. Biswas A. (2015). Metabolic risk factors of sporadic Alzheimer’s disease: Implications in the pathology, pathogenesis and treatment. *Aging Dis*. 6 282–299. 10.14336/ad.2014.002 26236550 PMC4509477

[B20] Chang S. L. Huang W. Mao X. Mack M. L. (2018). Ethanol’s effects on transient receptor potential channel expression in brain microvascular endothelial cells. *J. Neuroimmune Pharmacol*. 13 498–508. 10.1007/s11481-018-9796-3 29987591 PMC6431236

[B21] Chen C. C. Lu R. B. Chen Y. C. Wang M. F. Chang Y. C. Li T. K. (1999). Interaction between the functional polymorphisms of the alcohol-metabolism genes in protection against alcoholism. *Am. J. Hum. Genet*. 65 795–807. 10.1086/302540 10441588 PMC1377988

[B22] Chen C. H. Budas G. R. Churchill E. N. Disatnik M. H. Hurley T. D. Mochly-Rosen D. (2008). Activation of aldehyde dehydrogenase-2 reduces ischemic damage to the heart. *Science* 321 1493–1495. 10.1126/science.1158554 18787169 PMC2741612

[B23] Chen C. H. Ferreira J. C. B. Mochly-Rosen D. (2019). ALDH2 and Cardiovascular disease. *Adv. Exp. Med. Biol*. 1193 53–67. 10.1007/978-981-13-6260-6_3 31368097

[B24] Chen C. H. Joshi A. U. Mochly-Rosen D. (2016). The role of mitochondrial Aldehyde Dehydrogenase 2 (ALDH2) in neuropathology and neurodegeneration. *Acta Neurol. Taiwan* 25 111–123. PMC10618051 28382610

[B25] Chen C. H. Kraemer B. R. Mochly-Rosen D. (2022). ALDH2 variance in disease and populations. *Dis. Model. Mech*. 15:dmm049601. 10.1242/dmm.049601 35749303 PMC9235878

[B26] Chen F. Wang N. Tian X. Qin Y. Su J. He R. (2022). The potential diagnostic accuracy of urine formaldehyde levels in Alzheimer’s disease: A systematic review and meta-analysis. *Front. Aging Neurosci*. 14:1057059. 10.3389/fnagi.2022.1057059 36583189 PMC9794019

[B27] Chen K. Maley J. Yu P. H. (2006). Potential inplications of endogenous aldehydes in beta-amyloid misfolding, oligomerization and fibrillogenesis. *J. Neurochem*. 99 1413–1424. 10.1111/j.1471-4159.2006.04181.x 17074066

[B28] Chen Y. C. Peng G. S. Tsao T. P. Wang M. F. Lu R. B. Yin S. J. (2009). Pharmacokinetic and pharmacodynamic basis for overcoming acetaldehyde-induced adverse reaction in Asian alcoholics, heterozygous for the variant ALDH2*2 gene allele. *Pharmacogenet. Genomics* 19 588–599. 10.1097/FPC.0b013e32832ecf2e 19584771

[B29] Clayton D. A. Browning M. D. (2001). Deficits in the expression of the NR2B subunit in the hippocampus of aged Fisher 344 rats. *Neurobiol. Aging* 22 165–168. 10.1016/s0197-4580(00)00196-2 11164294

[B30] Clayton D. A. Mesches M. H. Alvarez E. Bickford P. C. Browning M. D. (2002). A hippocampal NR2B deficit can mimic age-related changes in long-term potentiation and spatial learning in the Fischer 344 rat. *J. Neurosci*. 22 3628–3637. 10.1523/jneurosci.22-09-03628.2002 11978838 PMC6758397

[B31] Crews F. T. Lawrimore C. J. Walter T. J. Coleman L. G. Jr. (2017). The role of neuroimmune signaling in alcoholism. *Neuropharmacology* 122 56–73. 10.1016/j.neuropharm.2017.01.031 28159648 PMC5493978

[B32] Dalleau S. Baradat M. Guéraud F. Huc L. (2013). Cell death and diseases related to oxidative stress: 4-Hydroxynonenal (HNE) in the balance. *Cell Death Differ*. 20 1615–1630. 10.1038/cdd.2013.138 24096871 PMC3824598

[B33] Di Domenico F. Tramutola A. Butterfield D. A. (2017). Role of 4-hydroxy-2-nonenal (HNE) in the pathogenesis of Alzheimer disease and other selected age-related neurodegenerative disorders. *Free Radic. Biol. Med*. 111 253–261. 10.1016/j.freeradbiomed.2016.10.490 27789292

[B34] Díaz G. Lengele L. Sourdet S. Soriano G. de Souto Barreto P. (2022). Nutrients and amyloid β status in the brain: A narrative review. *Ageing Res. Rev*. 81:101728. 10.1016/j.arr.2022.101728 36049590

[B35] Dingler F. A. Wang M. Mu A. Millington C. L. Oberbeck N. Watcham S. (2020). Two aldehyde clearance systems are essential to prevent lethal formaldehyde accumulation in mice and humans. *Mol. Cell*. 80 996–1012.e9. 10.1016/j.molcel.2020.10.012 33147438 PMC7758861

[B36] Drake J. Petroze R. Castegna A. Ding Q. Keller J. N. Markesbery W. R. (2004). 4-Hydroxynonenal oxidatively modifies histones: Implications for Alzheimer’s disease. *Neurosci. Lett*. 356 155–158. 10.1016/j.neulet.2003.11.047 15036618

[B37] D’Souza Y. Elharram A. Soon-Shiong R. Andrew R. D. Bennett B. M. (2015). Characterization of Aldh2 (-/-) mice as an age-related model of cognitive impairment and Alzheimer’s disease. *Mol. Brain* 8:27. 10.1186/s13041-015-0117-y 25910195 PMC4409701

[B38] Edenberg H. J. Foroud T. (2013). Genetics and alcoholism. *Nat. Rev. Gastroenterol. Hepatol*. 10 487–494. 10.1038/nrgastro.2013.86 23712313 PMC4056340

[B39] Eisele Y. S. Obermüller U. Heilbronner G. Baumann F. Kaeser S. A. Wolburg H. (2010). Peripherally applied Abeta-containing inoculates induce cerebral beta-amyloidosis. *Science* 330 980–982. 10.1126/science.1194516 20966215 PMC3233904

[B40] Elyaman W. Yardin C. Hugon J. (2002). Involvement of glycogen synthase kinase-3beta and tau phosphorylation in neuronal Golgi disassembly. *J. Neurochem*. 81 870–880. 10.1046/j.1471-4159.2002.00838.x 12065646

[B41] Fukuda M. Kanou F. Shimada N. Sawabe M. Saito Y. Murayama S. (2009). Elevated levels of 4-hydroxynonenal-histidine Michael adduct in the hippocampi of patients with Alzheimer’s disease. *Biomed. Res*. 30 227–233. 10.2220/biomedres.30.227 19729853

[B42] Griñan-Ferré C. Palomera-Ávalos V. Puigoriol-Illamola D. Camins A. Porquet D. Plá V. (2016). Behaviour and cognitive changes correlated with hippocampal neuroinflammaging and neuronal markers in female SAMP8, a model of accelerated senescence. *Exp. Gerontol*. 80 57–69. 10.1016/j.exger.2016.03.014 27094468

[B43] Griñán-Ferré C. Sarroca S. Ivanova A. Puigoriol-Illamola D. Aguado F. Camins A. (2016). Epigenetic mechanisms underlying cognitive impairment and Alzheimer disease hallmarks in 5XFAD mice. *Aging* 8 664–684. 10.18632/aging.100906 27013617 PMC4925821

[B44] Guo H. Yu X. Liu Y. Paik D. T. Justesen J. M. Chandy M. (2023). SGLT2 inhibitor ameliorates endothelial dysfunction associated with the common ALDH2 alcohol flushing variant. *Sci. Transl. Med*. 15:eab9952. 10.1126/scitranslmed.abp9952 36696485 PMC10297796

[B45] Gwon A. R. Park J. S. Arumugam T. V. Kwon Y. K. Chan S. L. Kim S. H. (2012). Oxidative lipid modification of nicastrin enhances amyloidogenic γ-secretase activity in Alzheimer’s disease. *Aging Cell* 11 559–568. 10.1111/j.1474-9726.2012.00817.x 22404891 PMC4217088

[B46] Haorah J. Knipe B. Leibhart J. Ghorpade A. Persidsky Y. (2005). Alcohol-induced oxidative stress in brain endothelial cells causes blood-brain barrier dysfunction. *J. Leukoc Biol*. 78 1223–1232. 10.1189/jlb.0605340 16204625

[B47] He R. Lu J. Miao J. (2010). Formaldehyde stress. *Sci. China Life Sci*. 53 1399–1404. 10.1007/s11427-010-4112-3 21181340

[B48] Hebert L. E. Weuve J. Scherr P. A. Evans D. A. (2013). Alzheimer disease in the United States (2010-2050) estimated using the 2010 census. *Neurology* 80 1778–1783. 10.1212/WNL.0b013e31828726f5 23390181 PMC3719424

[B49] Hoffman J. L. Faccidomo S. Kim M. Taylor S. M. Agoglia A. E. May A. M. (2019). Alcohol drinking exacerbates neural and behavioral pathology in the 3xTg-AD mouse model of Alzheimer’s disease. *Int Rev. Neurobiol*. 148 169–230. 10.1016/bs.irn.2019.10.017 31733664 PMC6939615

[B50] Hu Q. Teng W. Li J. Hao F. Wang N. (2016). Homocysteine and Alzheimer’s disease: Evidence for a causal link from mendelian randomization. *J. Alzheimers Dis*. 52 747–756. 10.3233/jad-150977 27031476

[B51] Huang H. Kozekov I. D. Kozekova A. Wang H. Lloyd R. S. Rizzo C. J. (2010). DNA cross-link induced by trans-4-hydroxynonenal. *Environ. Mol. Mutagen*. 51 625–634. 10.1002/em.20599 20577992 PMC3140422

[B52] Huang M. C. Tu H. Y. Chung R. H. Kuo H. W. Liu T. H. Chen C. H. (2023). Changes of neurofilament light chain in patients with alcohol dependence following withdrawal and the genetic effect from ALDH2 Polymorphism. *Eur. Arch. Psychiatry Clin. Neurosci*. 10.1007/s00406-023-01635-5 [Epub ahead of print]. 37314537 PMC10719424

[B53] Hynd M. R. Scott H. L. Dodd P. R. (2004). Differential expression of N-methyl-D-aspartate receptor NR2 isoforms in Alzheimer’s disease. *J. Neurochem*. 90 913–919. 10.1111/j.1471-4159.2004.02548.x 15287897

[B54] Ilomaki J. Jokanovic N. Tan E. C. Lonnroos E. (2015). Alcohol consumption, dementia and cognitive decline: An overview of systematic reviews. *Curr. Clin. Pharmacol*. 10 204–212. 10.2174/157488471003150820145539 26338173

[B55] Jeon K. H. Han K. Jeong S. M. Park J. Yoo J. E. Yoo J. (2023). Changes in alcohol consumption and risk of dementia in a nationwide cohort in South Korea. *JAMA Netw. Open* 6:e2254771. 10.1001/jamanetworkopen.2022.54771 36745453 PMC12549098

[B56] Jin X. Long T. Chen H. Zeng Y. Zhang X. Yan L. (2021). Associations of alcohol dehydrogenase and aldehyde dehydrogenase polymorphism with cognitive impairment among the oldest-old in China. *Front. Aging Neurosci*. 13:710966. 10.3389/fnagi.2021.710966 35368830 PMC8965653

[B57] Joshi A. U. Van Wassenhove L. D. Logas K. R. Minhas P. S. Andreasson K. I. Weinberg K. I. (2019). Aldehyde dehydrogenase 2 activity and aldehydic load contribute to neuroinflammation and Alzheimer’s disease related pathology. *Acta Neuropathol. Commun*. 7:190. 10.1186/s40478-019-0839-7 31829281 PMC6907112

[B58] Kamal H. Tan G. C. Ibrahim S. F. Shaikh M. F. Mohamed I. N. Mohamed R. M. P. (2020). Alcohol use disorder, neurodegeneration, Alzheimer’s and Parkinson’s Disease: Interplay between oxidative stress, neuroimmune response and excitotoxicity. *Front. Cell Neurosci*. 14:282. 10.3389/fncel.2020.00282 33061892 PMC7488355

[B59] Kamino K. Nagasaka K. Imagawa M. Yamamoto H. Yoneda H. Ueki A. (2000). Deficiency in mitochondrial aldehyde dehydrogenase increases the risk for late-onset Alzheimer’s disease in the Japanese population. *Biochem. Biophys. Res. Commun*. 273 192–196. 10.1006/bbrc.2000.2923 10873585

[B60] Kanamaru T. Kamimura N. Yokota T. Iuchi K. Nishimaki K. Takami S. (2015). Oxidative stress accelerates amyloid deposition and memory impairment in a double-transgenic mouse model of Alzheimer’s disease. *Neurosci. Lett*. 587 126–131. 10.1016/j.neulet.2014.12.033 25529196

[B61] Kawarabayashi T. Younkin L. H. Saido T. C. Shoji M. Ashe K. H. Younkin S. G. (2001). Age-dependent changes in brain, CSF, and plasma amyloid (beta) protein in the Tg2576 transgenic mouse model of Alzheimer’s disease. *J. Neurosci*. 21 372–381. 10.1523/jneurosci.21-02-00372.2001 11160418 PMC6763819

[B62] Keller J. N. Pang Z. Geddes J. W. Begley J. G. Germeyer A. Waeg G. (1997). Impairment of glucose and glutamate transport and induction of mitochondrial oxidative stress and dysfunction in synaptosomes by amyloid beta-peptide: Role of the lipid peroxidation product 4-hydroxynonenal. *J. Neurochem*. 69 273–284. 10.1046/j.1471-4159.1997.69010273.x 9202320

[B63] Keller J. N. Schmitt F. A. Scheff S. W. Ding Q. Chen Q. Butterfield D. A. (2005). Evidence of increased oxidative damage in subjects with mild cognitive impairment. *Neurology* 64 1152–1156. 10.1212/01.Wnl.0000156156.13641.Ba 15824339

[B64] Khan S. Barve K. H. Kumar M. S. (2020). Recent advancements in pathogenesis, diagnostics and treatment of Alzheimer’s disease. *Curr. Neuropharmacol*. 18 1106–1125. 10.2174/1570159x18666200528142429 32484110 PMC7709159

[B65] Kim J. M. Stewart R. Shin I. S. Jung J. S. Yoon J. S. (2004). Assessment of association between mitochondrial aldehyde dehydrogenase polymorphism and Alzheimer’s disease in an older Korean population. *Neurobiol. Aging* 25 295–301. 10.1016/s0197-4580(03)00114-3 15123334

[B66] Kim S. R. Jeong H. Y. Yang S. Choi S. P. Seo M. Y. Yun Y. K. (2011). Effects of chronic alcohol consumption on expression levels of APP and Aβ-producing enzymes. *BMB Rep*. 44 135–139. 10.5483/BMBRep.2011.44.2.135 21345314

[B67] Klyosov A. A. Rashkovetsky L. G. Tahir M. K. Keung W. M. (1996). Possible role of liver cytosolic and mitochondrial aldehyde dehydrogenases in acetaldehyde metabolism. *Biochemistry* 35 4445–4456. 10.1021/bi9521093 8605194

[B68] Knopp R. C. Lee S. H. Hollas M. Nepomuceno E. Gonzalez D. Tam K. (2020). Interaction of oxidative stress and neurotrauma in ALDH2(-/-) mice causes significant and persistent behavioral and pro-inflammatory effects in a tractable model of mild traumatic brain injury. *Redox Biol*. 32:101486. 10.1016/j.redox.2020.101486 32155582 PMC7063127

[B69] Koch M. Fitzpatrick A. L. Rapp S. R. Nahin R. L. Williamson J. D. Lopez O. L. (2019). Alcohol consumption and risk of dementia and cognitive decline among older adults with or without mild cognitive impairment. *JAMA Netw. Open* 2:e1910319. 10.1001/jamanetworkopen.2019.10319 31560382 PMC6777245

[B70] Komatsu M. Shibata N. Ohnuma T. Kuerban B. Tomson K. Toda A. (2014). Polymorphisms in the aldehyde dehydrogenase 2 and dopamine β hydroxylase genes are not associated with Alzheimer’s disease. *J. Neural Transm.* 121 427–432. 10.1007/s00702-013-1112-z 24201835

[B71] Kou Y. Zhao H. Cui D. Han H. Tong Z. (2022). Formaldehyde toxicity in age-related neurological dementia. *Ageing Res. Rev*. 73:101512. 10.1016/j.arr.2021.101512 34798299

[B72] Lam V. Takechi R. Hackett M. J. Francis R. Bynevelt M. Celliers L. M. (2021). Synthesis of human amyloid restricted to liver results in an Alzheimer disease-like neurodegenerative phenotype. *PLoS Biol*. 19:e3001358. 10.1371/journal.pbio.3001358 34520451 PMC8439475

[B73] Lane C. A. Hardy J. Schott J. M. (2018). Alzheimer’s disease. *Eur. J. Neurol*. 25 59–70. 10.1111/ene.13439 28872215

[B74] Lao Y. Hou L. Li J. Hui X. Yan P. Yang K. (2021). Association between alcohol intake, mild cognitive impairment and progression to dementia: A dose-response meta-analysis. *Aging Clin. Exp. Res*. 33 1175–1185. 10.1007/s40520-020-01605-0 32488474

[B75] León B. E. Kang S. Franca-Solomon G. Shang P. Choi D. S. (2021). Alcohol-induced neuroinflammatory response and mitochondrial dysfunction on aging and Alzheimer’s disease. *Front. Behav. Neurosci*. 15:778456. 10.3389/fnbeh.2021.778456 35221939 PMC8866940

[B76] Li M. H. Tang J. P. Zhang P. Li X. Wang C. Y. Wei H. J. (2014). Disturbance of endogenous hydrogen sulfide generation and endoplasmic reticulum stress in hippocampus are involved in homocysteine-induced defect in learning and memory of rats. *Behav. Brain Res*. 262 35–41. 10.1016/j.bbr.2014.01.001 24423987

[B77] Li M. Zhang P. Wei H. J. Li M. H. Zou W. Li X. (2017). Hydrogen sulfide ameliorates homocysteine-induced cognitive dysfunction by inhibition of reactive aldehydes involving upregulation of ALDH2. *Int. J. Neuropsychopharmacol*. 20 305–315. 10.1093/ijnp/pyw103 27988490 PMC5409037

[B78] Li T. Wei Y. Qu M. Mou L. Miao J. Xi M. (2021). Formaldehyde and De/Methylation in age-related cognitive impairment. *Genes* 12:913. 10.3390/genes12060913 34199279 PMC8231798

[B79] Li X. Chen W. Huang X. Jing W. Zhang T. Yu Q. (2021). Synaptic dysfunction of Aldh1a1 neurons in the ventral tegmental area causes impulsive behaviors. *Mol. Neurodegener*. 16:73. 10.1186/s13024-021-00494-9 34702328 PMC8549305

[B80] Liu L. Komatsu H. Murray I. V. Axelsen P. H. (2008). Promotion of amyloid beta protein misfolding and fibrillogenesis by a lipid oxidation product. *J. Mol. Biol*. 377 1236–1250. 10.1016/j.jmb.2008.01.057 18304576

[B81] Livingston G. Huntley J. Sommerlad A. Ames D. Ballard C. Banerjee S. (2020). Dementia prevention, intervention, and care: 2020 report of the Lancet Commission. *Lancet* 396 413–446. 10.1016/s0140-6736(20)30367-6 32738937 PMC7392084

[B82] Lok K. Zhao H. Shen H. Wang Z. Gao X. Zhao W. (2013). Characterization of the APP/PS1 mouse model of Alzheimer’s disease in senescence accelerated background. *Neurosci. Lett*. 557(Pt. B) 84–89. 10.1016/j.neulet.2013.10.051 24176881

[B83] Lovell M. A. Ehmann W. D. Mattson M. P. Markesbery W. R. (1997). Elevated 4-hydroxynonenal in ventricular fluid in Alzheimer’s disease. *Neurobiol. Aging* 18 457–461. 10.1016/s0197-4580(97)00108-5 9390770

[B84] Lu J. Miao J. Su T. Liu Y. He R. (2013). Formaldehyde induces hyperphosphorylation and polymerization of Tau protein both in vitro and in vivo. *Biochim. Biophys. Acta* 1830 4102–4116. 10.1016/j.bbagen.2013.04.028 23628704

[B85] Luo J. Lee S. H. VandeVrede L. Qin Z. Ben Aissa M. Larson J. (2016). A multifunctional therapeutic approach to disease modification in multiple familial mouse models and a novel sporadic model of Alzheimer’s disease. *Mol. Neurodegener*. 11:35. 10.1186/s13024-016-0103-6 27129593 PMC4850651

[B86] Ma L. Lu Z. N. (2016). Role of ADH1B rs1229984 and ALDH2 rs671 gene polymorphisms in the development of Alzheimer’s disease. *Genet. Mol. Res*. 15. 10.4238/gmr.15048740 27808372

[B87] Manthey J. Shield K. D. Rylett M. Hasan O. S. M. Probst C. Rehm J. (2019). Global alcohol exposure between 1990 and 2017 and forecasts until 2030: A modelling study. *Lancet* 393 2493–2502. 10.1016/s0140-6736(18)32744-2 31076174

[B88] Marchitti S. A. Brocker C. Stagos D. Vasiliou V. (2008). Non-P450 aldehyde oxidizing enzymes: The aldehyde dehydrogenase superfamily. *Expert Opin. Drug Metab. Toxicol*. 4 697–720. 10.1517/17425255.4.6.697 18611112 PMC2658643

[B89] Mark R. J. Lovell M. A. Markesbery W. R. Uchida K. Mattson M. P. (1997). A role for 4-hydroxynonenal, an aldehydic product of lipid peroxidation, in disruption of ion homeostasis and neuronal death induced by amyloid beta-peptide. *J. Neurochem*. 68 255–264. 10.1046/j.1471-4159.1997.68010255.x 8978733

[B90] Markesbery W. R. Lovell M. A. (1998). Four-hydroxynonenal, a product of lipid peroxidation, is increased in the brain in Alzheimer’s disease. *Neurobiol. Aging* 19 33–36. 10.1016/s0197-4580(98)00009-8 9562500

[B91] Masters C. L. Bateman R. Blennow K. Rowe C. C. Sperling R. A. Cummings J. L. (2015). Alzheimer’s disease. *Nat. Rev. Dis. Primers* 1:15056. 10.1038/nrdp.2015.56 27188934

[B92] Mehder R. H. Bennett B. M. Andrew R. D. (2020). Morphometric analysis of hippocampal and neocortical pyramidal neurons in a mouse model of late onset Alzheimer’s disease. *J. Alzheimers Dis*. 74 1069–1083. 10.3233/jad-191067 32144984 PMC7242838

[B93] Mehder R. H. Bennett B. M. Andrew R. D. (2021). Age-related neuronal deterioration specifically within the dorsal CA1 region of the hippocampus in a mouse model of late onset Alzheimer’s disease. *J. Alzheimers Dis*. 79 1547–1561. 10.3233/jad-201024 33459722 PMC7990463

[B94] Mesches M. H. Gemma C. Veng L. M. Allgeier C. Young D. A. Browning M. D. (2004). Sulindac improves memory and increases NMDA receptor subunits in aged Fischer 344 rats. *Neurobiol. Aging* 25 315–324. 10.1016/s0197-4580(03)00116-7 15123337

[B95] Meza V. Arnold J. Díaz L. A. Ayala Valverde M. Idalsoaga F. Ayares G. (2022). Alcohol consumption: Medical implications, the liver and beyond. *Alcohol Alcohol*. 57 283–291. 10.1093/alcalc/agac013 35333295

[B96] Miller C. A. Gavin C. F. White J. A. Parrish R. R. Honasoge A. Yancey C. R. (2010). Cortical DNA methylation maintains remote memory. *Nat. Neurosci*. 13 664–666. 10.1038/nn.2560 20495557 PMC3043549

[B97] Morales R. Duran-Aniotz C. Bravo-Alegria J. Estrada L. D. Shahnawaz M. Hu P. P. (2020). Infusion of blood from mice displaying cerebral amyloidosis accelerates amyloid pathology in animal models of Alzheimer’s disease. *Acta Neuropathol. Commun*. 8:213. 10.1186/s40478-020-01087-1 33287898 PMC7720397

[B98] Mu A. Hira A. Niwa A. Osawa M. Yoshida K. Mori M. (2021). Analysis of disease model iPSCs derived from patients with a novel Fanconi anemia-like IBMFS ADH5/ALDH2 deficiency. *Blood* 137 2021–2032. 10.1182/blood.2020009111 33512438

[B99] Nannelli G. Terzuoli E. Giorgio V. Donnini S. Lupetti P. Giachetti A. (2018). ALDH2 activity reduces mitochondrial oxygen reserve capacity in endothelial cells and induces senescence properties. *Oxid. Med. Cell Longev*. 2018:9765027. 10.1155/2018/9765027 30538807 PMC6261243

[B100] Nannelli G. Ziche M. Donnini S. Morbidelli L. (2020). Endothelial aldehyde dehydrogenase 2 as a target to maintain vascular wellness and function in ageing. *Biomedicines* 8:4. 10.3390/biomedicines8010004 31947800 PMC7168060

[B101] Nie C. L. Wang X. S. Liu Y. Perrett S. He R. Q. (2007a). Amyloid-like aggregates of neuronal tau induced by formaldehyde promote apoptosis of neuronal cells. *BMC Neurosci*. 8:9. 10.1186/1471-2202-8-9 17241479 PMC1790706

[B102] Nie C. L. Wei Y. Chen X. Liu Y. Y. Dui W. Liu Y. (2007b). Formaldehyde at low concentration induces protein tau into globular amyloid-like aggregates in vitro and in vivo. *PLoS One* 2:e629. 10.1371/journal.pone.0000629 17637844 PMC1913207

[B103] Nikhil K. Viccaro K. Shah K. (2019). Multifaceted regulation of ALDH1A1 by Cdk5 in Alzheimer’s disease pathogenesis. *Mol. Neurobiol*. 56 1366–1390. 10.1007/s12035-018-1114-9 29948941 PMC6368892

[B104] Obermeier B. Daneman R. Ransohoff R. M. (2013). Development, maintenance and disruption of the blood-brain barrier. *Nat. Med*. 19 1584–1596. 10.1038/nm.3407 24309662 PMC4080800

[B105] Ohsawa I. Nishimaki K. Murakami Y. Suzuki Y. Ishikawa M. Ohta S. (2008). Age-dependent neurodegeneration accompanying memory loss in transgenic mice defective in mitochondrial aldehyde dehydrogenase 2 activity. *J. Neurosci*. 28 6239–6249. 10.1523/jneurosci.4956-07.2008 18550766 PMC6670537

[B106] Ohsawa I. Nishimaki K. Yasuda C. Kamino K. Ohta S. (2003). Deficiency in a mitochondrial aldehyde dehydrogenase increases vulnerability to oxidative stress in PC12 cells. *J. Neurochem*. 84 1110–1117. 10.1046/j.1471-4159.2003.01619.x 12603834

[B107] Ohta S. Ohsawa I. Kamino K. Ando F. Shimokata H. (2004). Mitochondrial ALDH2 deficiency as an oxidative stress. *Ann. N. Y. Acad. Sci*. 1011 36–44. 10.1007/978-3-662-41088-2_4 15126281

[B108] Ormeño D. Romero F. López-Fenner J. Avila A. Martínez-Torres A. Parodi J. (2013). Ethanol reduces amyloid aggregation in vitro and prevents toxicity in cell lines. *Arch. Med. Res*. 44 1–7. 10.1016/j.arcmed.2012.12.004 23291379

[B109] Owen J. B. Sultana R. Aluise C. D. Erickson M. A. Price T. O. Bu G. (2010). Oxidative modification to LDL receptor-related protein 1 in hippocampus from subjects with Alzheimer disease: Implications for Aβ accumulation in AD brain. *Free Radic. Biol. Med*. 49 1798–1803. 10.1016/j.freeradbiomed.2010.09.013 20869432 PMC2970765

[B110] Padurariu M. Ciobica A. Lefter R. Serban I. L. Stefanescu C. Chirita R. (2013). The oxidative stress hypothesis in Alzheimer’s disease. *Psychiatr. Danub*. 25 401–409. 24247053

[B111] Parodi J. Ormeño D. Ochoa-de la Paz L. D. (2015). Amyloid pore-channel hypothesis: Effect of ethanol on aggregation state using frog oocytes for an Alzheimer’s disease study. *BMB Rep*. 48 13–18. 10.5483/bmbrep.2015.48.1.125 25047445 PMC4345636

[B112] Peng B. Yang Q. B Joshi R. Liu Y. Akbar M. Song B. J. (2020). Role of alcohol drinking in Alzheimer’s disease, Parkinson’s disease, and amyotrophic lateral sclerosis. *Int. J. Mol. Sci*. 21:2316. 10.3390/ijms21072316 32230811 PMC7177420

[B113] Perluigi M. Coccia R. Butterfield D. A. (2012). 4-Hydroxy-2-nonenal, a reactive product of lipid peroxidation, and neurodegenerative diseases: A toxic combination illuminated by redox proteomics studies. *Antioxid. Redox Signal*. 17 1590–1609. 10.1089/ars.2011.4406 22114878 PMC3449441

[B114] Perluigi M. Sultana R. Cenini G. Di Domenico F. Memo M. Pierce W. M. (2009). Redox proteomics identification of 4-hydroxynonenal-modified brain proteins in Alzheimer’s disease: Role of lipid peroxidation in Alzheimer’s disease pathogenesis. *Proteom. Clin. Appl*. 3 682–693. 10.1002/prca.200800161 20333275 PMC2843938

[B115] Pimentel E. Sivalingam K. Doke M. Samikkannu T. (2020). Effects of drugs of abuse on the blood-brain barrier: A brief overview. *Front. Neurosci*. 14:513. 10.3389/fnins.2020.00513 32670001 PMC7326150

[B116] Piumatti G. Moore S. C. Berridge D. M. Sarkar C. Gallacher J. (2018). The relationship between alcohol use and long-term cognitive decline in middle and late life: A longitudinal analysis using UK Biobank. *J. Public Health* 40 304–311. 10.1093/pubmed/fdx186 29325150 PMC6051452

[B117] Praticò D. (2008). Oxidative stress hypothesis in Alzheimer’s disease: A reappraisal. *Trends Pharmacol. Sci*. 29 609–615. 10.1016/j.tips.2008.09.001 18838179

[B118] Praticò D. Uryu K. Leight S. Trojanoswki J. Q. Lee V. M. (2001). Increased lipid peroxidation precedes amyloid plaque formation in an animal model of Alzheimer amyloidosis. *J. Neurosci*. 21 4183–4187. 10.1523/jneurosci.21-12-04183.2001 11404403 PMC6762743

[B119] Rajgopal Y. Vemuri M. C. (2001). Ethanol induced changes in cyclin-dependent kinase-5 activity and its activators, P35, P67 (Munc-18) in rat brain. *Neurosci. Lett*. 308 173–176. 10.1016/s0304-3940(01)02011-0 11479016

[B120] Ramos A. Joshi R. S. Szabo G. (2022). Innate immune activation: Parallels in alcohol use disorder and Alzheimer’s disease. *Front. Mol. Neurosci*. 15:910298. 10.3389/fnmol.2022.910298 36157070 PMC9505690

[B121] Ran L. S. Liu W. H. Fang Y. Y. Xu S. B. Li J. Luo X. (2021). Alcohol, coffee and tea intake and the risk of cognitive deficits: A dose-response meta-analysis. *Epidemiol. Psychiatr. Sci*. 30:e13. 10.1017/s2045796020001183 33568254 PMC8061189

[B122] Reale M. Costantini E. Jagarlapoodi S. Khan H. Belwal T. Cichelli A. (2020). Relationship of wine consumption with Alzheimer’s disease. *Nutrients* 12:206. 10.3390/nu12010206 31941117 PMC7019227

[B123] Reed T. T. Pierce W. M. Markesbery W. R. Butterfield D. A. (2009). Proteomic identification of HNE-bound proteins in early Alzheimer disease: Insights into the role of lipid peroxidation in the progression of AD. *Brain Res*. 1274 66–76. 10.1016/j.brainres.2009.04.009 19374891

[B124] Resende R. Moreira P. I. Proença T. Deshpande A. Busciglio J. Pereira C. (2008). Brain oxidative stress in a triple-transgenic mouse model of Alzheimer disease. *Free Radic. Biol. Med*. 44 2051–2057. 10.1016/j.freeradbiomed.2008.03.012 18423383

[B125] Rodríguez-Zavala J. S. Calleja L. F. Moreno-Sánchez R. Yoval-Sánchez B. (2019). Role of aldehyde dehydrogenases in physiopathological processes. *Chem. Res. Toxicol*. 32 405–420. 10.1021/acs.chemrestox.8b00256 30628442

[B126] Roy B. Palaniyandi S. S. (2020). Aldehyde dehydrogenase 2 inhibition potentiates 4-hydroxy-2-nonenal induced decrease in angiogenesis of coronary endothelial cells. *Cell Biochem. Funct*. 38 290–299. 10.1002/cbf.3468 31943249

[B127] Roy B. Sundar K. Palaniyandi S. S. (2020). 4-hydroxy-2-nonenal decreases coronary endothelial cell migration: Potentiation by aldehyde dehydrogenase 2 inhibition. *Vascul. Pharmacol*. 131:106762. 10.1016/j.vph.2020.106762 32585188

[B128] Rubio-Araiz A. Porcu F. Pérez-Hernández M. García-Gutiérrez M. S. Aracil-Fernández M. A. Gutierrez-López M. D. (2017). Disruption of blood-brain barrier integrity in postmortem alcoholic brain: Preclinical evidence of TLR4 involvement from a binge-like drinking model. *Addict. Biol*. 22 1103–1116. 10.1111/adb.12376 26949123

[B129] Rumgay H. Shield K. Charvat H. Ferrari P. Sornpaisarn B. Obot I. (2021). Global burden of cancer in 2020 attributable to alcohol consumption: A population-based study. *Lancet Oncol*. 22 1071–1080. 10.1016/s1470-2045(21)00279-5 34270924 PMC8324483

[B130] Sabia S. Fayosse A. Dumurgier J. Dugravot A. Akbaraly T. Britton A. (2018). Alcohol consumption and risk of dementia: 23 year follow-up of Whitehall II cohort study. *BMJ* 362:k2927. 10.1136/bmj.k2927 30068508 PMC6066998

[B131] Sayre L. M. Zelasko D. A. Harris P. L. Perry G. Salomon R. G. Smith M. A. (1997). 4-Hydroxynonenal-derived advanced lipid peroxidation end products are increased in Alzheimer’s disease. *J. Neurochem*. 68 2092–2097. 10.1046/j.1471-4159.1997.68052092.x 9109537

[B132] Scheltens P. De Strooper B. Kivipelto M. Holstege H. Chételat G. Teunissen C. E. (2021). Alzheimer’s disease. *Lancet* 397 1577–1590. 10.1016/s0140-6736(20)32205-4 33667416 PMC8354300

[B133] Schneider J. A. Arvanitakis Z. Bang W. Bennett D. A. (2007). Mixed brain pathologies account for most dementia cases in community-dwelling older persons. *Neurology* 69 2197–2204. 10.1212/01.wnl.0000271090.28148.24 17568013

[B134] Schwarzinger M. Pollock B. G. Hasan O. S. M. Dufouil C. Rehm J. (2018). Contribution of alcohol use disorders to the burden of dementia in France 2008-13: A nationwide retrospective cohort study. *Lancet Public Health* 3 e124–e132. 10.1016/s2468-2667(18)30022-7 29475810

[B135] Seike T. Boontem P. Yanagi M. Li S. Kido H. Yamamiya D. (2022). Hydroxynonenal causes hepatocyte death by disrupting lysosomal integrity in nonalcoholic steatohepatitis. *Cell Mol. Gastroenterol. Hepatol*. 14 925–944. 10.1016/j.jcmgh.2022.06.008 35787976 PMC9500440

[B136] Seike T. Mizukoshi E. Kaneko S. (2021). Role of CD4+ T-cells in the pathology of non-alcoholic fatty liver disease and related diseases. *Hepatoma Res*. 7:46. 10.20517/2394-5079.2021.46

[B137] Seike T. Mizukoshi E. Yamada K. Okada H. Kitahara M. Yamashita T. (2020). Fatty acid-driven modifications in T-cell profiles in non-alcoholic fatty liver disease patients. *J. Gastroenterol*. 55 701–711. 10.1007/s00535-020-01679-7 32124081

[B138] Seshadri S. Beiser A. Selhub J. Jacques P. F. Rosenberg I. H. D’Agostino R. B. (2002). Plasma homocysteine as a risk factor for dementia and Alzheimer’s disease. *N. Engl. J. Med*. 346 476–483. 10.1056/NEJMoa011613 11844848

[B139] Shimizu E. Tang Y. P. Rampon C. Tsien J. Z. (2000). NMDA receptor-dependent synaptic reinforcement as a crucial process for memory consolidation. *Science* 290 1170–1174. 10.1126/science.290.5494.1170 11073458

[B140] Shin I. S. Stewart R. Kim J. M. Kim S. W. Yang S. J. Shin H. Y. (2005). Mitochondrial aldehyde dehydrogenase polymorphism is not associated with incidence of Alzheimer’s disease. *Int. J. Geriatr. Psychiatry* 20 1075–1080. 10.1002/gps.1401 16250071

[B141] Shin S. W. Kim D. H. Jeon W. K. Han J. S. (2020). 4-hydroxynonenal immunoreactivity is increased in the frontal cortex of 5XFAD transgenic mice. *Biomedicines* 8:326. 10.3390/biomedicines8090326 32899155 PMC7554765

[B142] Shringarpure R. Grune T. Sitte N. Davies K. J. (2000). 4-Hydroxynonenal-modified amyloid-beta peptide inhibits the proteasome: Possible importance in Alzheimer’s disease. *Cell Mol. Life Sci*. 57 1802–1809. 10.1007/pl00000660 11130184 PMC11149552

[B143] Siegel S. J. Bieschke J. Powers E. T. Kelly J. W. (2007). The oxidative stress metabolite 4-hydroxynonenal promotes Alzheimer protofibril formation. *Biochemistry* 46 1503–1510. 10.1021/bi061853s 17279615 PMC2530822

[B144] Singh A. K. Jiang Y. Gupta S. Benlhabib E. (2007). Effects of chronic ethanol drinking on the blood brain barrier and ensuing neuronal toxicity in alcohol-preferring rats subjected to intraperitoneal LPS injection. *Alcohol Alcohol*. 42 385–399. 10.1093/alcalc/agl120 17341516

[B145] Spence J. P. Liang T. Eriksson C. J. Taylor R. E. Wall T. L. Ehlers C. L. (2003). Evaluation of aldehyde dehydrogenase 1 promoter polymorphisms identified in human populations. *Alcohol Clin. Exp. Res*. 27 1389–1394. 10.1097/01.Alc.0000087086.50089.59 14506398 PMC4560114

[B146] Sultana R. Perluigi M. Butterfield D. A. (2013). Lipid peroxidation triggers neurodegeneration: A redox proteomics view into the Alzheimer disease brain. *Free Radic. Biol. Med*. 62 157–169. 10.1016/j.freeradbiomed.2012.09.027 23044265 PMC3573239

[B147] Sweeney M. D. Sagare A. P. Zlokovic B. V. (2018). Blood-brain barrier breakdown in Alzheimer disease and other neurodegenerative disorders. *Nat. Rev. Neurol*. 14 133–150. 10.1038/nrneurol.2017.188 29377008 PMC5829048

[B148] Tamagno E. Bardini P. Obbili A. Vitali A. Borghi R. Zaccheo D. (2002). Oxidative stress increases expression and activity of BACE in NT2 neurons. *Neurobiol. Dis*. 10 279–288. 10.1006/nbdi.2002.0515 12270690

[B149] Tamagno E. Parola M. Bardini P. Piccini A. Borghi R. Guglielmotto M. (2005). Beta-site APP cleaving enzyme up-regulation induced by 4-hydroxynonenal is mediated by stress-activated protein kinases pathways. *J. Neurochem*. 92 628–636. 10.1111/j.1471-4159.2004.02895.x 15659232

[B150] Tan T. Zhang Y. Luo W. Lv J. Han C. Hamlin J. N. R. (2018). Formaldehyde induces diabetes-associated cognitive impairments. *FASEB J*. 32 3669–3679. 10.1096/fj.201701239R 29401634

[B151] Tandon R. Levey A. I. Lah J. J. Seyfried N. T. Mitchell C. S. (2023). Machine learning selection of most predictive brain proteins suggests role of sugar metabolism in Alzheimer’s disease. *J. Alzheimers Dis*. 92 411–424. 10.3233/jad-220683 36776048 PMC10041447

[B152] Tao R. Liao M. Wang Y. Wang H. Tan Y. Qin S. (2022). In situ imaging of formaldehyde in live mice with high spatiotemporal resolution reveals aldehyde dehydrogenase-2 as a potential target for Alzheimer’s disease treatment. *Anal. Chem*. 94 1308–1317. 10.1021/acs.analchem.1c04520 34962779

[B153] Teng S. Beard K. Pourahmad J. Moridani M. Easson E. Poon R. (2001). The formaldehyde metabolic detoxification enzyme systems and molecular cytotoxic mechanism in isolated rat hepatocytes. *Chem. Biol. Interact*. 130-132 285–296. 10.1016/s0009-2797(00)00272-6 11306052

[B154] Tong Z. Han C. Luo W. Li H. Luo H. Qiang M. (2013a). Aging-associated excess formaldehyde leads to spatial memory deficits. *Sci. Rep*. 3:1807. 10.1038/srep01807 23657727 PMC3648839

[B155] Tong Z. Han C. Luo W. Wang X. Li H. Luo H. (2013b). Accumulated hippocampal formaldehyde induces age-dependent memory decline. *Age* 35 583–596. 10.1007/s11357-012-9388-8 22382760 PMC3636394

[B156] Tong Z. Han C. Qiang M. Wang W. Lv J. Zhang S. (2015). Age-related formaldehyde interferes with DNA methyltransferase function, causing memory loss in Alzheimer’s disease. *Neurobiol. Aging* 36 100–110. 10.1016/j.neurobiolaging.2014.07.018 25282336

[B157] Tong Z. Zhang J. Luo W. Wang W. Li F. Li H. (2011). Urine formaldehyde level is inversely correlated to mini mental state examination scores in senile dementia. *Neurobiol. Aging* 32 31–41. 10.1016/j.neurobiolaging.2009.07.013 19879019

[B158] Topiwala A. Allan C. L. Valkanova V. Zsoldos E. Filippini N. Sexton C. (2017). Moderate alcohol consumption as risk factor for adverse brain outcomes and cognitive decline: Longitudinal cohort study. *BMJ* 357:j2353. 10.1136/bmj.j2353 28588063 PMC5460586

[B159] Ueno M. Yoshino Y. Mori H. Funahashi Y. Kumon H. Ochi S. (2022). Association study and meta-analysis of polymorphisms and blood mRNA expression of the ALDH2 gene in patients with Alzheimer’s disease. *J. Alzheimers Dis*. 87 863–871. 10.3233/jad-215627 35404279 PMC9198735

[B160] Van Dam F. Van Gool W. A. (2009). Hyperhomocysteinemia and Alzheimer’s disease: A systematic review. *Arch. Gerontol. Geriatr*. 48 425–430. 10.1016/j.archger.2008.03.009 18479766

[B161] Venkataraman A. Kalk N. Sewell G. Ritchie C. Lingford-Hughes A. (2017). Alcohol and Alzheimer’s disease-does alcohol dependence contribute to beta-amyloid deposition, neuroinflammation and neurodegeneration in Alzheimer’s disease? *Alcohol Alcohol*. 52:158. 10.1093/alcalc/agw101 28182204

[B162] Visontay R. Rao R. T. Mewton L. (2021). Alcohol use and dementia: New research directions. *Curr. Opin. Psychiatry* 34 165–170. 33394727 10.1097/YCO.0000000000000679

[B163] Wang B. Wang J. Zhou S. Tan S. He X. Yang Z. (2008). The association of mitochondrial aldehyde dehydrogenase gene (ALDH2) polymorphism with susceptibility to late-onset Alzheimer’s disease in Chinese. *J. Neurol. Sci*. 268 172–175. 10.1016/j.jns.2007.12.006 18201725

[B164] Wang D. S. Iwata N. Hama E. Saido T. C. Dickson D. W. (2003). Oxidized neprilysin in aging and Alzheimer’s disease brains. *Biochem. Biophys. Res. Commun*. 310 236–241. 10.1016/j.bbrc.2003.09.003 14511676

[B165] Wang J. Gu B. J. Masters C. L. Wang Y. J. (2017). A systemic view of Alzheimer disease - insights from amyloid-β metabolism beyond the brain. *Nat. Rev. Neurol*. 13 612–623. 10.1038/nrneurol.2017.111 28960209

[B166] Wang R. Wang S. Malter J. S. Wang D. S. (2009). Effects of HNE-modification induced by Abeta on neprilysin expression and activity in SH-SY5Y cells. *J. Neurochem*. 108 1072–1082. 10.1111/j.1471-4159.2008.05855.x 19196432 PMC2693391

[B167] Westerman M. A. Cooper-Blacketer D. Mariash A. Kotilinek L. Kawarabayashi T. Younkin L. H. (2002). The relationship between Abeta and memory in the Tg2576 mouse model of Alzheimer’s disease. *J. Neurosci*. 22 1858–1867. 10.1523/jneurosci.22-05-01858.2002 11880515 PMC6758862

[B168] Wiegmann C. Mick I. Brandl E. J. Heinz A. Gutwinski S. (2020). Alcohol and dementia - what is the link? A systematic review. *Neuropsychiatr. Dis. Treat*. 16 87–99. 10.2147/ndt.S198772 32021202 PMC6957093

[B169] Williams T. I. Lynn B. C. Markesbery W. R. Lovell M. A. (2006). Increased levels of 4-hydroxynonenal and acrolein, neurotoxic markers of lipid peroxidation, in the brain in Mild Cognitive Impairment and early Alzheimer’s disease. *Neurobiol. Aging* 27 1094–1099. 10.1016/j.neurobiolaging.2005.06.004 15993986

[B170] Wu K. M. Zhang Y. R. Huang Y. Y. Dong Q. Tan L. Yu J. T. (2021). The role of the immune system in Alzheimer’s disease. *Ageing Res. Rev*. 70:101409. 10.1016/j.arr.2021.101409 34273589

[B171] Wu Y. Y. Lee Y. S. Liu Y. L. Hsu W. C. Ho W. M. Huang Y. H. (2021). Association study of alcohol dehydrogenase and aldehyde dehydrogenase polymorphism with Alzheimer disease in the Taiwanese population. *Front. Neurosci*. 15:625885. 10.3389/fnins.2021.625885 33551739 PMC7862325

[B172] Xu F. Chen Y. G. Xue L. Li R. J. Zhang H. Bian Y. (2011). Role of aldehyde dehydrogenase 2 Glu504lys polymorphism in acute coronary syndrome. *J. Cell Mol. Med*. 15 1955–1962. 10.1111/j.1582-4934.2010.01181.x 21958412 PMC3918050

[B173] Xu W. Wang H. Wan Y. Tan C. Li J. Tan L. (2017). Alcohol consumption and dementia risk: A dose-response meta-analysis of prospective studies. *Eur. J. Epidemiol*. 32 31–42. 10.1007/s10654-017-0225-3 28097521

[B174] Xue L. Yang F. Han Z. Cui S. Dai S. Xu F. (2018). ALDH2 mediates the dose-response protection of chronic ethanol against endothelial senescence through SIRT1/p53 pathway. *Biochem. Biophys. Res. Commun*. 504 777–783. 10.1016/j.bbrc.2018.08.081 30217444

[B175] Xue L. Zhu W. Yang F. Dai S. Han Z. Xu F. (2019). Appropriate dose of ethanol exerts anti-senescence and anti-atherosclerosis protective effects by activating ALDH2. *Biochem. Biophys. Res. Commun*. 512 319–325. 30885430 10.1016/j.bbrc.2019.03.037

[B176] Yamashima T. Boontem P. Kido H. Yanagi M. Seike T. (2022). Hydroxynonenal causes lysosomal and autophagic failure in the monkey POMC neurons. *J. Alzheimers Dis. Parkinson*. 12 2161–2460.

[B177] Yamashima T. Ota T. Mizukoshi E. Nakamura H. Yamamoto Y. Kikuchi M. (2020). Intake of ω-6 polyunsaturated fatty acid-rich vegetable oils and risk of lifestyle diseases. *Adv. Nutr*. 11 1489–1509. 10.1093/advances/nmaa072 32623461 PMC7666899

[B178] Yamashima T. Seike T. Oikawa S. Kobayashi H. Kido H. Yanagi M. (2023). Hsp70.1 carbonylation induces lysosomal cell death for lifestyle-related diseases. *Front. Mol. Biosci.* 9:1063632. 10.3389/fmolb.2022.1063632 36819480 PMC9936620

[B179] Yang Y. Chen W. Wang X. Ge W. (2021). Impact of mitochondrial aldehyde dehydrogenase 2 on cognitive impairment in the AD model mouse. *Acta Biochim. Biophys. Sin.* 53 837–847. 10.1093/abbs/gmab057 33954430

[B180] Yasue H. Mizuno Y. Harada E. (2019). Association of East Asian variant aldehyde dehydrogenase 2 genotype (ALDH2*2*) with coronary spasm and acute myocardial infarction. *Adv. Exp. Med. Biol*. 1193 121–134. 10.1007/978-981-13-6260-6_7 31368101

[B181] Yokoyama A. Kato H. Yokoyama T. Tsujinaka T. Muto M. Omori T. (2002). Genetic polymorphisms of alcohol and aldehyde dehydrogenases and glutathione S-transferase M1 and drinking, smoking, and diet in Japanese men with esophageal squamous cell carcinoma. *Carcinogenesis* 23 1851–1859. 10.1093/carcin/23.11.1851 12419833

[B182] Yokoyama A. Tsutsumi E. Imazeki H. Suwa Y. Nakamura C. Mizukami T. (2008). Salivary acetaldehyde concentration according to alcoholic beverage consumed and aldehyde dehydrogenase-2 genotype. *Alcohol Clin. Exp. Res*. 32 1607–1614. 10.1111/j.1530-0277.2008.00739.x 18616675

[B183] Yoshida A. Davé V. Ward R. J. Peters T. J. (1989). Cytosolic aldehyde dehydrogenase (ALDH1) variants found in alcohol flushers. *Ann. Hum. Genet*. 53 1–7. 10.1111/j.1469-1809.1989.tb01116.x 2729894

[B184] Yue X. Mei Y. Zhang Y. Tong Z. Cui D. Yang J. (2019). New insight into Alzheimer’s disease: Light reverses Aβ-obstructed interstitial fluid flow and ameliorates memory decline in APP/PS1 mice. *Alzheimers Dement.* 5 671–684. 10.1016/j.trci.2019.09.007 31720368 PMC6838540

[B185] Zhang C. E. Wei W. Liu Y. H. Peng J. H. Tian Q. Liu G. P. (2009). Hyperhomocysteinemia increases beta-amyloid by enhancing expression of gamma-secretase and phosphorylation of amyloid precursor protein in rat brain. *Am. J. Pathol*. 174 1481–1491. 10.2353/ajpath.2009.081036 19264913 PMC2671378

[B186] Zhang H. Fu L. (2021). The role of ALDH2 in tumorigenesis and tumor progression: Targeting ALDH2 as a potential cancer treatment. *Acta Pharm. Sin. B*. 11 1400–1411. 10.1016/j.apsb.2021.02.008 34221859 PMC8245805

[B187] Zhang J. Yue X. Luo H. Jiang W. Mei Y. Ai L. (2019). Illumination with 630 nm red light reduces oxidative stress and restores memory by photo-activating catalase and formaldehyde dehydrogenase in SAMP8 mice. *Antioxid. Redox Signal*. 30 1432–1449. 10.1089/ars.2018.7520 29869529

[B188] Zhang P. Edenberg H. J. Nurnberger J. Lai D. Cheng F. Liu Y. (2022). Alcohol use disorder is associated with higher risks of Alzheimer’s and Parkinson’s diseases: A study of US insurance claims data. *Alzheimers Dement.* 14:e12370. 10.1002/dad2.12370 36419637 PMC9677510

[B189] Zhang R. Shen L. Miles T. Shen Y. Cordero J. Qi Y. (2020). Association of low to moderate alcohol drinking with cognitive functions from middle to older age among US adults. *JAMA Netw. Open* 3:e207922. 10.1001/jamanetworkopen.2020.7922 32597992 PMC7324954

[B190] Zhou S. Huriletemuer Wang J. Zhang C. Zhao S. Wang de S. (2010). Absence of association on aldehyde dehydrogenase 2 (ALDH2) polymorphism with Mongolian Alzheimer patients. *Neurosci. Lett*. 468 312–315. 10.1016/j.neulet.2009.11.022 19914339

[B191] Zhu X. Raina A. K. Perry G. Smith M. A. (2004). Alzheimer’s disease: The two-hit hypothesis. *Lancet Neurol*. 3 219–226. 10.1016/s1474-4422(04)00707-0 15039034

[B192] Zhu Z. Y. Liu Y. D. Gong Y. Jin W. Topchiy E. Turdi S. (2022). Mitochondrial aldehyde dehydrogenase (ALDH2) rescues cardiac contractile dysfunction in an APP/PS1 murine model of Alzheimer’s disease via inhibition of ACSL4-dependent ferroptosis. *Acta Pharmacol. Sin*. 43 39–49. 10.1038/s41401-021-00635-2 33767380 PMC8724276

[B193] Zilkens R. R. Bruce D. G. Duke J. Spilsbury K. Semmens J. B. (2014). Severe psychiatric disorders in mid-life and risk of dementia in late- life (age 65-84 years): A population based case-control study. *Curr. Alzheimer Res*. 11 681–693. 10.2174/1567205011666140812115004 25115541 PMC4153082

